# A virus-acquired host cytokine controls systemic aging by antagonizing apoptosis

**DOI:** 10.1371/journal.pbio.2005796

**Published:** 2018-07-23

**Authors:** Mohamed Mlih, Mobina Khericha, Christine Birdwell, A. Phillip West, Jason Karpac

**Affiliations:** 1 Dept. of Molecular and Cellular Medicine, Texas A&M University Health Science Center, College Station, Texas, United States of America; 2 Dept. of Microbial Pathogenesis and Immunology, Texas A&M University Health Science Center, College Station, Texas, United States of America; The Francis Crick Institute, United Kingdom of Great Britain and Northern Ireland

## Abstract

Aging is characterized by degeneration of unique tissues. However, dissecting the interconnectedness of tissue aging remains a challenge. Here, we employ a muscle-specific DNA damage model in *Drosophila* to reveal secreted factors that influence systemic aging in distal tissues. Utilizing this model, we uncovered a cytokine—Diedel—that, when secreted from muscle or adipose, can attenuate age-related intestinal tissue degeneration by promoting proliferative homeostasis of stem cells. Diedel is both necessary and sufficient to limit tissue degeneration and regulate lifespan. Secreted homologs of Diedel are also found in viruses, having been acquired from host genomes. Focusing on potential mechanistic overlap between cellular aging and viral-host cell interactions, we found that Diedel is an inhibitor of apoptosis and can act as a systemic rheostat to modulate cell death during aging. These results highlight a key role for secreted antagonists of apoptosis in the systemic coordination of tissue aging.

## Author summary

Aging in multicellular organisms is characterized by a progressive decline in the proper function of organs. This deterioration of organ function is a risk factor for many diseases. However, it is unlikely that organs age in isolation, as damage in one organ can presumably impact aging of other organs through either beneficial or detrimental cross-talk. Our work attempts to explore this aspect of aging using fruit flies as a model system. We uncovered that damaged fly muscle can protect against aging in other organs, such as the intestine, through the secretion of a blood-borne factor named Diedel. This blood-borne factor presumably allows damaged organs to communicate with each other during aging. Related factors are also found in certain viruses, which have been hijacked from insect genomes to promote viral spreading during infection. Using this information, we found that viral Diedel inhibits death of infected cells, allowing viruses to spread. Similarly, host (insect) Diedel also blocks cell death in organs during aging, thus limiting deterioration of organ function and extending the organism’s lifespan.

## Introduction

In most metazoans, aging is characterized by a drastic decline in cellular and tissue function. This ultimate decline in function is driven by a multitude of complex, age-related cellular changes. The cellular and tissue changes that promote aging can be defined by (i) primary causes of cellular damage (such as genomic instability/DNA damage and telomere attrition), (ii) antagonistic responses to damage (such as changes in mitochondria function, cell death regulation/senescence, and changes in nutrient sensing), and (iii) integrative responses that eventually promote tissue dysfunction (such as a decline in regenerative potential, described in detail in [1]). Adding to this complexity is the fact that tissues do not age in isolation, as aging also involves changes at the level of intertissue communication. The interconnectedness of primary, antagonistic, and integrative responses in the context of tissue aging is likely to involve both autonomous and systemic mechanisms. This is highlighted by the systemic control of age-related changes in tissue regeneration.

Attenuation in regenerative potential is a defining characteristic of aged tissues and a primary driver of tissue dysfunction. This decline in regenerative potential originates from age-related changes in the regulation of stem cell function, which include stem cell exhaustion and/or deficient proliferation and differentiation, as well as stem cell hyperproliferation [1–3]. Collectively, a loss of proliferative homeostasis within stem and progenitor cells during aging promotes tissue dysfunction and degeneration. Heterochronic parabiosis and heterochronic blood exchange experiments have intricately demonstrated that the systemic environment plays an immense role in controlling age-related changes in proliferative homeostasis [2]. Systemic or secreted factors from “old blood” can have inhibitory effects on young tissue stem cells, and although less robust, “young blood” can reverse the decline of stem cell function in aged tissues [4–6].

The precise nature of communication between unique tissues with diverse functions, during aging, remains unclear. However, the systemic coordination of tissue aging and regeneration, or the ability of age-related changes in one tissue to alter aging in distal tissues, is constricted by both deleterious and beneficial consequences.

Antagonistic mechanisms that respond to primary causes of age-related cellular damage also govern proliferative homeostasis to control tissue regeneration. This includes altered mitochondrial function and associated stress response pathways (such as the endoplasmic reticulum stress response [ER stress], the unfolded protein response [UPR], mitochondrial DNA [mtDNA] stress, and autophagy), cell senescence, altered programmed cell death/apoptosis, and changes in nutrient sensing (through insulin-like growth factor [IGF], insulin, and target of rapamycin [TOR] signaling), [7–13]. Critically, many of these signaling mechanisms and stress pathways promote both normal tissue regeneration and age-related tissue degeneration, highlighting the pleiotropic nature of antagonistic responses to cellular aging. Thus, uncovering secreted factors (such as cytokines, growth factors, and other small molecules) that both enable organ cross-talk and precisely modulate antagonistic responses to affect proliferative homeostasis is likely to reveal fundamental mechanisms driving the systemic coordination of tissue aging and longevity.

The utilization of these antagonistic stress response mechanisms is not limited to cell-autonomous/nonautonomous control of tissue function and regeneration. Similar signaling mechanisms play critical roles in pathogen-host cell interactions, especially those related to viruses. For example, host cells can employ mtDNA stress, ER/UPR stress, cell senescence, and/or apoptotic responses as antiviral mechanisms [14–17]. In contrast, many viruses have developed mechanisms to suppress or modify these host responses to facilitate viral replication and spread. The coevolution of viruses and hosts has also promoted viral hijacking of host-secreted factors (encoded virokines) in order to alter cell signaling and cell communication to favor virus-infected cells [18–20]. The obvious mechanistic overlap between cellular aging and pathogen–host cell interactions suggests that pathogens may contain vital information associated with age-related changes in intercellular or intertissue communication.

To this end, we show here that a secreted cytokine named Diedel, which has also been acquired by viruses [21, 22], is a critical regulator of systemic tissue aging. Utilizing the genetic capacity of *Drosophila*, we designed a model in order to characterize age-related systemic interactions between distal tissues that influence proliferative homeostasis, tissue degeneration, and longevity (described in detail in the section Hormetic responses from muscle-specific DNA damage attenuate systemic tissue aging and enhance lifespan). Using this fly model, we uncovered that Diedel, when secreted from muscle or adipose, can attenuate age-related intestinal tissue degeneration by promoting proliferative homeostasis of stem cells and that Diedel is both necessary and sufficient to limit tissue degeneration and extend lifespan. Focusing on potential mechanistic overlap between cellular aging and viral-host cell interactions, we found that Diedel and its viral homolog act as functionally conserved inhibitors of apoptosis. For the virus, virokine Diedel can limit apoptosis to facilitate viral abundance and spread. For the host, cytokine Diedel acts as a systemic rheostat to limit apoptosis-induced degeneration of the aging intestine. These results highlight a key role for secreted antagonists of apoptosis, specifically those acquired by viral genomes, in the systemic coordination of tissue aging.

## Results

### Hormetic responses from muscle-specific DNA damage attenuate systemic tissue aging and enhance lifespan

In *Drosophila*, genetic manipulations in multiple individual tissues and unique cell types have been shown to impact longevity. In regard to systemic aging, muscle and fat body (adipose) appear to be critical effector/modulator tissues, and the brain can function as a control center though the regulation of secreted hormones [23–26]. The fly intestine is also central to lifespan. The *Drosophila* adult midgut (intestine) is regenerated by a population of intestinal stem cells (ISCs), and in a young midgut, ISCs divide rarely [27–29]. However, during aging, changes in activity of a multitude of stress response pathways leads to stem cell hyperproliferation/misdifferentiation and epithelial dysplasia [30]. This results in tissue degeneration, which ultimately limits lifespan [31].

**Fig 1:**
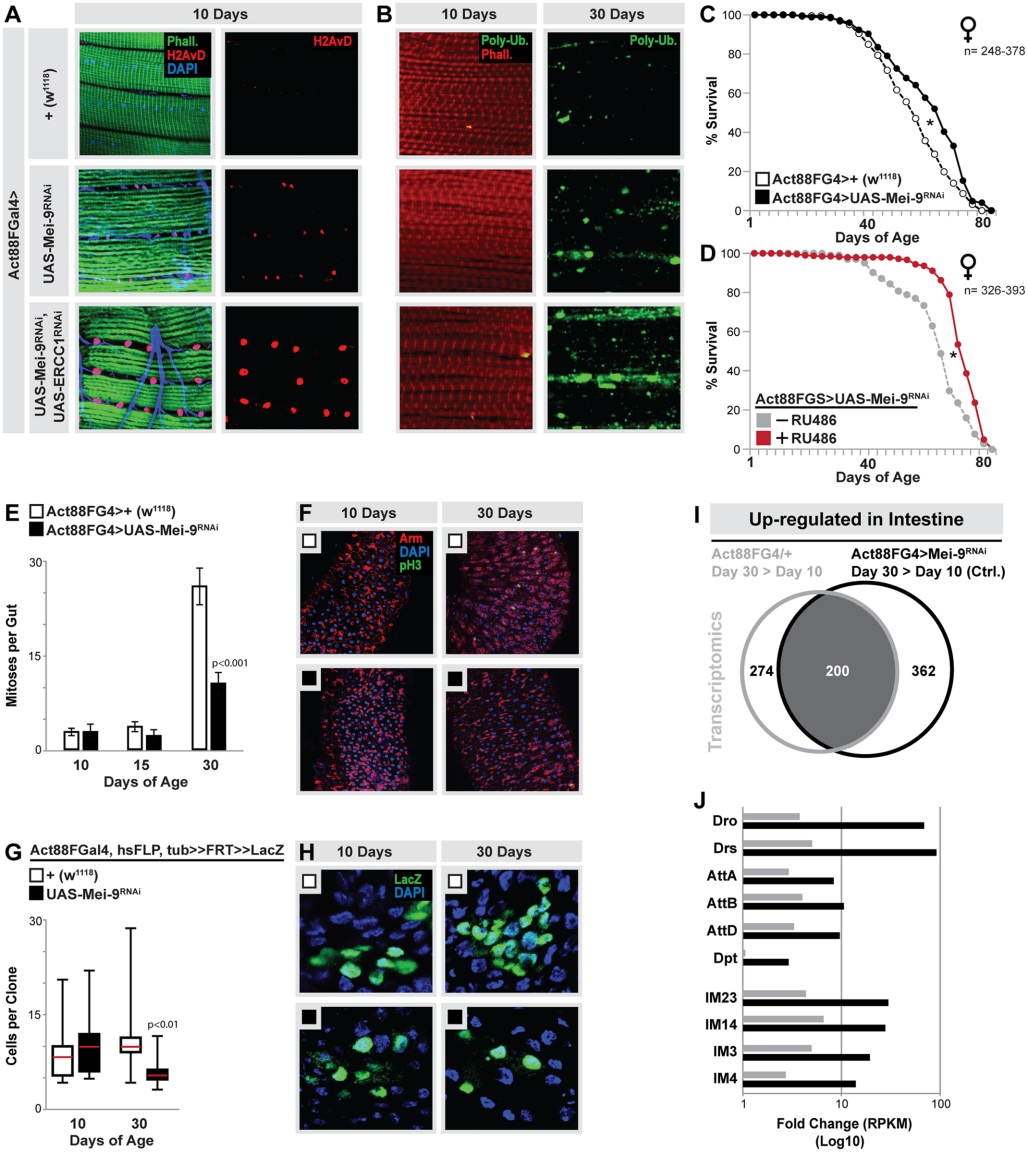
Systemic hormetic responses from muscle-specific DNA damage. (A) Detection of DNA damage (double strand breaks) in dissected longitudinal thoracic muscle of young (10 d) Act88FG4>+(w^1118^) controls, and flies with DNA repair attenuation specifically in thoracic muscle (mu-specific, Act88FG4>UAS-Mei-9^RNAi^ or Act88FG4>UAS-Mei-9^RNAi^, UAS-ERCC1^RNAi^); assayed by phospho-H2aV immunostaining (red), counterstained with phalloidin (“Phall”; green, actin filaments) and DAPI (blue). Representative images shown. (B) Immunostaining to detect poly-ubiquitin protein (aggregates; “Poly-Ub.”) in dissected longitudinal thoracic muscle from young (10 d) and old (30 d) flies, genotypes described above; anti-poly-ubiquitin (green), counterstained with phalloidin (red, actin filaments). Representative images shown. (C-D). Survival curves (lifespan, female flies) associated with mu-specific inhibition of Mei-9 using (C) the Act88FGal4 driver (compared to Act88FG4>+[w^1118^] controls) or (D) a GeneSwitch inducible driver (Act88FGS, +RU486 compared with −RU486 [vehicle alone] sibling controls). (E) Quantification of mitoses per whole dissected midgut (assayed by anti-pH3 immunostaining) at indicted ages, genotypes described above; bars represent mean ± SE, *n* = 25–30. (F) Immunostaining of dissected intestines to assess epithelial integrity of posterior midguts at indicated ages, genotypes described above; pH3 (green), armadillo (“Arm”; membrane, red), and DAPI (blue). Representative images shown. (G-H) Lineage tracing from ISCs using FRT recombination of a split *alpha-tubulin-lacZ* transgene (in Act88FG4>+[w^1118^, controls] or Act88FG4>UAS-Mei-9^RNAi^ genetic background). (G) Changes in clone size (cell per clone form posterior midgut) at indicated ages; represented as box plot (median, red line), *n* = 25. (H) Representative images of lacZ clones from various genotypes at indicated ages, immunostaining of dissected midguts (posterior), anti-lacZ (green), and DAPI (blue). (I) Venn diagrams showing overlap of up-regulated genes (from dissected midguts) between Act88FG4>UAS-Mei-9^RNAi^ and controls Act88FGal4>+(w^1118^) during aging (transcriptomes at 30 d, compared to Act88FGal4>+[w^1118^] controls at day 10). The threshold for genes included in the analysis was (i) changes in RPKM values of at least 2-fold up-regulated in intestine compared to young controls and (ii) a minimum RPKM value of 2. (J) Fold change (in intestinal transcriptome RPKM values; Day 30 Act88FG4>UAS-Mei-9^RNAi^/ Day 10 Act88FGal4>+[w^1118^] control [black bars] or Day 30 control/Day 10 control [gray bars]) of selected innate immune genes. Underlying data can be found in S1 Data. See also S1 and S2 Figs and S1 and S2 Tables. FRT, flippase recombination target; ISC, intestinal stem cell; pH3, phospho Histone H3; mu-specific, muscle-specific; RPKM, reads per kbp per million reads; RU486, mifepristone.

In order to uncover novel systemic mechanisms and communication axes that govern age-related changes in proliferative homeostasis, we tested multiple genetic strategies in order to induce tissue-specific and age-dependent tissue damage/stress within thoracic skeletal muscle, with the aim to assay integrative responses to primary causes of aging in distal tissues. The muscle has emerged as a critical regulator of longevity in *Drosophila*, and muscle injury in mammals has been suggested to influence regeneration in distal organs [24, 32, 33]. We discovered that mild attenuation of DNA repair within skeletal muscle leads to systemic responses that limit age-related intestinal degeneration and extend lifespan. Using RNA interference (RNAi)-mediated targeting of the Mei-9-ERCC1 heterodimeric DNA repair nuclease (S1 Fig and [34, 35]) specifically in adult thoracic skeletal muscle (using Act88FGal4, active in longitudinal indirect flight muscle but absent from other muscle found in the carcass and intestine [36]), we found that inhibiting DNA repair can dose- and age-dependently promote DNA damage, ultimately leading to muscle that presents hallmarks of tissue damage and premature aging (loss of proteostasis, Fig 1A and 1B). Attenuating Mei-9 alone (Act88FGal4>UAS-Mei-9^RNAi^) results in mild up-regulation of DNA damage and age-related increases in markers of muscle tissue damage/stress when compared to controls (Act88FGal4>+[w^1118^], Fig 1A and 1B and S1C Fig). Despite these subtle changes in muscle aging, there was no indication of profound damage to the tissue, in regard to muscle structure, function, or feeding behavior (previously linked to muscle aging, Fig 1A and 1B and S4A and S4B Fig). However, concurrently attenuating both Mei-9 and ERCC1 (Act88FGal4>UAS-Mei-9^RNAi^, UAS-ERCC1^RNAi^) leads to robust increases in both DNA damage and apparent tissue damage, highlighted by a premature loss of proteostasis and disruption of myofibril muscle structure during aging (Fig 1A and 1B).

Beneficial effects of adaptive responses to low doses of cellular stress or damage (termed hormesis) have been linked to systemic aging, including stress associated with genomic instability [37, 38]. We found that mild increases in muscle DNA damage (Act88FGal4>UAS-Mei-9^RNAi^) also increase mean adult lifespan and decrease age-specific mortality, although the impact on mortality rate was slightly reduced later in life (Fig 1C, S2A Fig and S1 Table). In order to confirm these longevity results, we generated a thoracic muscle-specific GeneSwitch driver (Act88FGS) for adult-specific and mifepristone (RU486)-inducible gene expression (S1E–S1L Fig). Inhibiting DNA repair with this driver extends mean lifespan as well (Act88FGS>UAS-Mei-9^RNAi^, Fig 1D, S3A and S3B Fig and S2 Table). Muscle-specific DNA damage can also systemically attenuate intestinal tissue degeneration during aging. Inhibiting Mei-9 function in muscle limits age-related ISC hyperproliferation and epithelial dysplasia (Fig 1E and 1F). Utilizing a split-lacZ reconstitution assay to heritably label-select ISCs (clones), we also found that muscle-specific DNA damage limits clone growth (cells per clone) in aged midguts (Fig 1G and 1H), further highlighting a preservation of proliferative homeostasis. Female flies are used exclusively for all assays (except for survival analysis) because of sexual dimorphism related to midgut regeneration [39]. Alternatively, strong enrichment of muscle-specific DNA damage (Act88FGal4>UAS-Mei-9^RNAi^, UAS-ERCC1^RNAi^) does not positively impact age-related tissue degeneration or lifespan (S4C and S4D Fig and S2 Table), and inhibiting DNA repair in other tissues, such as directly in the midgut, leads to accelerated aging (S4E–S4G Fig and S1 Table). These results highlight the dose dependency and tissue specificity associated with DNA damage–mediated hormesis.

Taken together, our data show that mild enhancement of DNA damage in muscle can drive systemic hormetic responses to promote proliferative homeostasis of distal regenerating tissues during aging and increase longevity.

### Cytokine Diedel governs systemic tissue aging and lifespan

We next wanted to use this genetic model of hormesis, and putative muscle-gut communication axis, to uncover systemic factors/mechanisms that modulate age-related changes in proliferative homeostasis. To this end, we generated genome-wide expression profiles from dissected thoraces (enriched in indirect flight muscle) and intestines of young and old flies with muscle-specific attenuation of DNA repair (S4H and S4I Fig). In midguts, we found a significant overlap in age-related intestinal transcriptome changes (focusing on up-regulated gene expression) between Act88FGal4>UAS-Mei-9^RNAi^ flies and controls (Fig 1I), despite the strong rescue of intestinal epithelial dysplasia in animals with Mei-9 inhibition in muscle. Gene Ontology (GO) clustering analysis revealed that the reason for this overlap is due to overrepresentation of GO terms associated with innate immune responses. Chronic activation of innate immunity in the *Drosophila* intestine during aging can promote tissue degeneration [40], and age-related up-regulation of innate genes occurs in both genotypes (select genes highlighted in Fig 1J and confirmed by quantitative real-time PCR [qRT-PCR] in S4J and S4K Fig). These data suggest that the maintenance of proliferative homeostasis in flies with muscle-specific DNA damage is not due to a general suppression of age-related systemic inflammatory responses and is likely achieved through more direct mechanisms.

**Fig 2:**
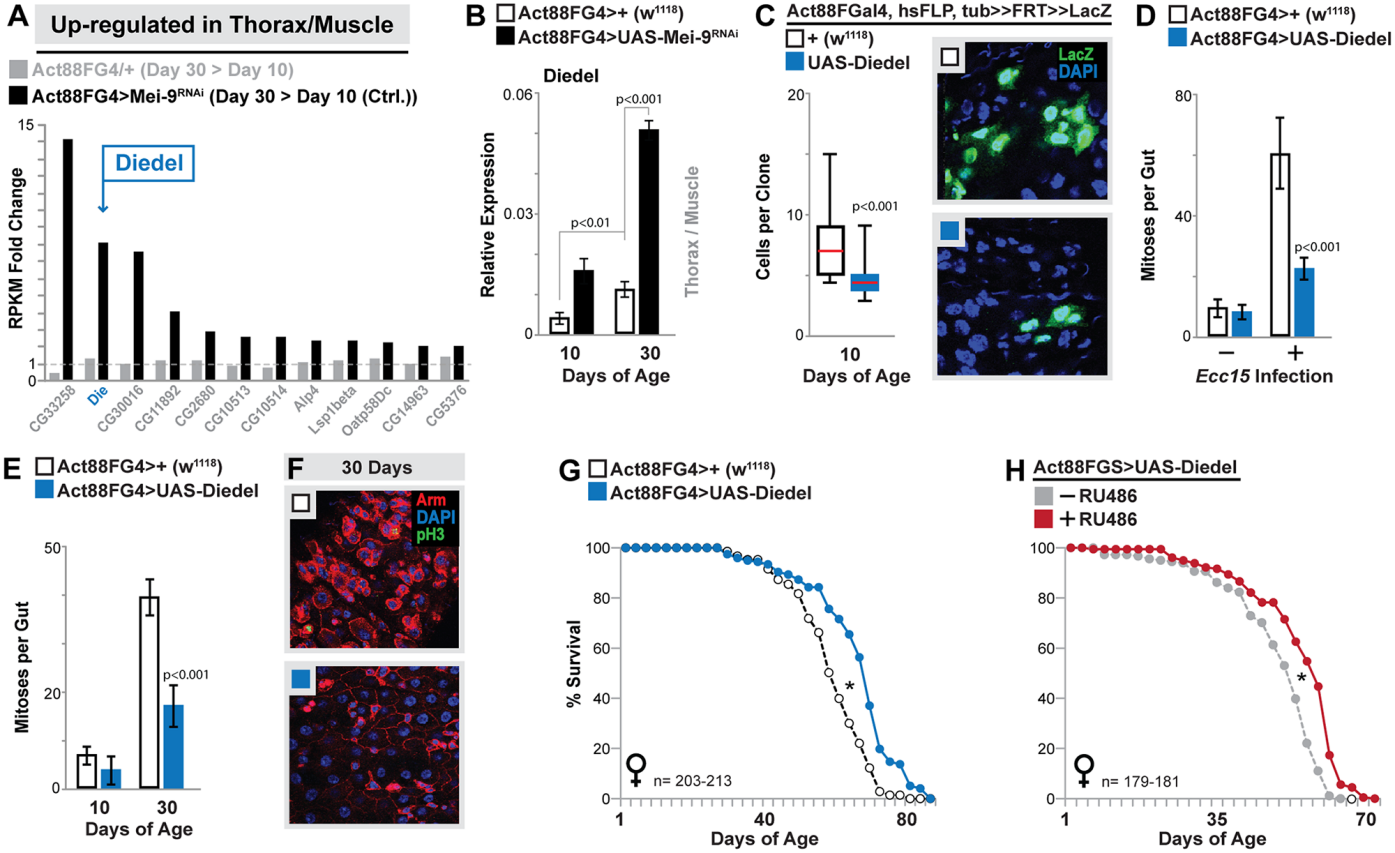
Muscle-derived Diedel attenuates systemic intestinal tissue aging and extends lifespan. (A) Histogram plotting fold change (in thorax/muscle transcriptome RPKM values of genes at least 2-fold up-regulated in Day 30 Act88FGal4>Mei-9^RNAi^/Day 10 Act88FGal4>+(w^1118^) control samples, but unchanged in control aging thorax/muscle samples. Diedel (“Die”; blue arrow). (B) Diedel transcription (measured by qRT-PCR) in dissected thorax/muscle upon mu-specific inhibition of Mei-9 at indicated ages; bars represent mean ± SE, *n* = 6. (C) Lineage tracing from ISCs using FRT recombination of a split *alpha-tubulin-lacZ* transgene (in Act88FG4>+[w^1118^, controls] or Act88FG4>UAS-Diedel genetic background); changes in clone size (cell per clone from posterior midgut) at Day 10; represented as box plot (median, red line), *n* = 25; Representative images of lacZ clones from various genotypes, immunostaining of dissected midguts (posterior), anti-lacZ (green), and DAPI (blue). (D-E) Quantification of mitoses per whole dissected midgut after (D) *Ecc15* oral infection (Day 10) or (E) during aging (assayed by anti-pH3 immunostaining) at indicated ages in Act88FG4>UAS-Diedel and control (Act88FG4>+[w^1118^]) flies; bars represent mean ± SE, *n* = 20–30. (F) Immunostaining of dissected intestines to assess epithelial integrity of posterior midguts at indicated ages, genotypes described above; pH3 (green), armadillo (“Arm”; membrane, red), and DAPI (blue). Representative images shown. (G-H) Survival curves (lifespan, female flies) associated with mu-specific expression of Diedel using (G) the Act88FGal4 driver (compared to Act88FG4>+[w^1118^] controls) or (H) a GeneSwitch inducible driver (Act88FGS, +RU486 compared to −RU486 [vehicle alone] sibling controls). Underlying data can be found in S1 Data. See also S1 and S2 Figs and S1 and S2 Tables. *Ecc15*, *E*. *carotovora carotovora 15*; FRT, flippase recombination target; ISC, intestinal stem cell; mu-specific, muscle-specific; qRT-PCR, quantitative real-time PCR; pH3, phospho Histone H3; RPKM, reads per kbp per million reads; RU486, mifepristone.

We turned to thoracic/muscle transcriptomes in order to identify genes or secreted factors the might mediate muscle–gut communication during aging. We discovered a small subset of genes that are up-regulated uniquely in aged Act88FGal4>UAS-Mei-9^RNAi^ thorax/muscle samples (with minimal or no induction in controls samples, Fig 2A). One gene in particular, named Diedel, was of distinct interest because it encodes a small protein with a putative signal peptide, and a recent report has shown that Diedel is a secreted factor (cytokine) found in circulation (hemolymph, [21]). Gene expression profiles in *Drosophila* have previously linked Diedel transcriptional induction to sterile injury, septic infection, viral infection, and tissue damage [21, 41–46]. In our model, Diedel expression is strongly up-regulated in Act88FGal4>UAS-Mei-9^RNAi^ thorax/muscle (and also slightly up-regulated in controls) during aging (Fig 2B). We thus asked whether Diedel expression from muscle could phenocopy the systemic hormetic responses induced by muscle-specific DNA repair inhibition. Indeed, overexpression of transgenic Diedel cDNA in skeletal muscle (Act88FGal4>UAS-Diedel, S5A Fig) affects both intestinal tissue regeneration and aging. Diedel expression in muscle limits ISC-derived clone growth (Fig 2C) in young animals, as well as regenerative responses (ISC proliferation) induced by natural infection of enteropathogenic bacteria (*Erwinia carotovora carotovora 15* [*Ecc*15], Fig 2D). Furthermore, muscle-specific Diedel expression also inhibits age-related ISC hyperproliferation and epithelial dysplasia (Fig 2E and 2F) and increases mean lifespan (utilizing both Gal4 and GeneSwitch systems, Fig 2G and 2H, S2B and S3C Figs, S1 and S2 Tables).

**Fig 3:**
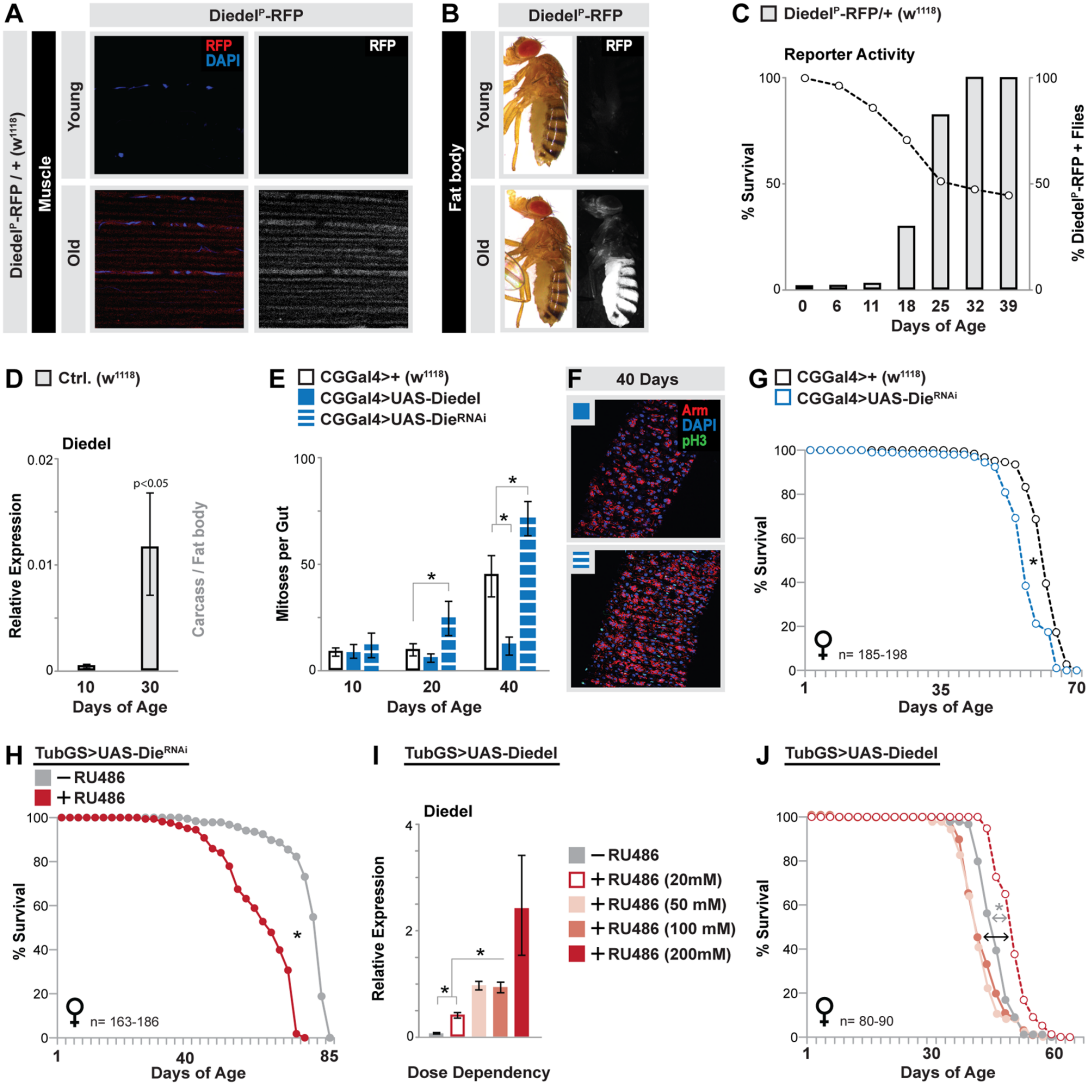
Diedel is necessary and sufficient to limit intestinal tissue degeneration and extend lifespan. (A-B) Images of Diedel reporter flies (Diedel^P^-RFP/+[w^1118^]) that express RFP under the control of the Diedel promoter. (A) RFP-fluorescence images of young (10 d) and old (30 d) dissected thoracic longitudinal muscle. (B) RFP-fluorescence images of young (10 d) and old (30 d) flies showing activation of the Diedel promoter in the abdominal fat body. Images represent Diedel^P^-RFP + flies. (C) Survival curves (open circles) of Diedel^P^-RFP/+(w^1118^) flies (*n* = 100 per population) plotted together with changes in the proportion of RFP+ flies in the population during aging (gray bars) at indicated ages. One of multiple experiments. (D) Diedel transcription (measured by qRT-PCR) in dissected carcass/fat body from young (10 d) and old (30 d) Diedel^P^-RFP/+(w^1118^) flies; bars represent mean ± SE, *n* = 4. (E) Quantification of mitoses per whole dissected midgut (assayed by anti-pH3 immunostaining) at indicted ages from CGG4>UAS-Diedel, CGG4>UAS-Die^RNAi^, and ctrls. (CGG4>+[w^1118^]); bars represent mean ± SE, *n* = 25–30, *p-value < 0.01. (F) Immunostaining of dissected intestines to assess epithelial integrity of posterior midguts at Day 40, genotypes described above; pH3 (green), armadillo (“Arm”; membrane, red), and DAPI (blue). Representative images shown. (G-H) Survival curves (lifespan, female flies) associated with Diedel inhibition (RNAi) using (C) the fat body driver CGG4 (compared to CGG4>+[w^1118^] controls) or (D) a ubiquitous GeneSwitch inducible driver (Tubulin(“Tub”)GS, +RU486 compared to −RU486 [vehicle alone] sibling ctrls.). (I) Diedel transcription (in TubGS>UAS-Diedel flies) measured by qRT-PCR in whole flies upon feeding different concentrations of RU486; after eclosion, flies were fed RU486 food for 5 d; bars represent mean ± SE, *n* = 3, *p-value < 0.01. (J) RU486 dose–dependency of lifespan in TubGS>UAS-Diedel female flies, utilizing 20 mM, 50 mM, 100 mM, and vehicle (-RU486) doses. Underlying data can be found in S1 Data. See also S3 Fig and S1, S2 and S3 Tables. Ctrl., control; pH3, phospho Histone H3; qRT-PCR, quantitative real-time PCR; RFP, red fluorescent protein; RNAi, RNA interference; RU486, mifepristone.

These data thus suggest that Diedel is an important systemic factor governing tissue aging and longevity. Because this gene can be induced by a wide variety of cellular and tissue stress (some related to aging), we wanted to determine if Diedel is important for normal aging, independent of our hormetic model. Surprisingly, Diedel knock-down in muscle (using RNAi-mediated targeting of the Diedel, UAS-Die^RNAi^) had no impact on systemic intestinal tissue aging (S5B and S5C Fig). We hypothesized that Diedel was either not required for normal aging or may be expressed in multiple tissues during aging. To answer this question, we generated Diedel reporter flies (using a 366-base-pair region located upstream of Diedel transcription start site, linked to red fluorescent protein [RFP]; Diedel^P^-RFP) to monitor Diedel expression changes in aging flies. Diedel reporter activity is off in young animals, but during aging, mild reporter activity is found in the muscle, while strong activation is observed in carcass fat body (muscle and fat body activation appear concurrently in the same animal, Fig 3A and 3B). Populations show a significant age-dependent increase in Diedel^P^-RFP animals, reaching nearly 100% of surviving flies later in life (Fig 3C). Similar affects are seen in our model of hormesis. DNA repair attenuation in muscle leads to reporter activation in the carcass fat body, although at a much accelerated rate over controls (S5D Fig), and age-related changes in Diedel gene expression are even stronger in fat body (Fig 3D and S5E Fig) when compared with the thorax/muscle (Fig 2B). Muscle tissue stress is thus likely to autonomously and nonautonomously (in fat body) promote Diedel expression and secretion [41].

Unlike the muscle, Diedel expression in fat body is required for normal systemic tissue aging and longevity. Diedel knock-down in fat body (using CGGal4 and PplGal4 coupled with RNAi) leads to accelerated aging of the intestine, in regard to ISC hyperproliferation and epithelial dysplasia (Fig 3E and 3F and S5F Fig). Similar to muscle, overexpression of Diedel in fat body also attenuates the age-related loss of proliferative homeostasis in the midgut (Fig 3E) and does not impact feeding behavior (S5G Fig). Diedel is also required for normal longevity, as inhibiting Diedel in fat body or ubiquitously in the adult animal (using the TubGeneSwitch driver) shortens fly lifespan and increases age-specific mortality (Fig 3G and 3H, S3D Fig, S1 and S2 Tables). Ubiquitous or fat body–specific overexpression of Diedel also shortens lifespan, despite the attenuation of intestinal dysplasia (S1 and S2 Tables). Previous reports have shown that the balance between normal regeneration and dysplasia in this tissue determines longevity [31]. Furthermore, Diedel overexpression using the muscle-specific GeneSwitch driver does not obviously impact mortality rates, notwithstanding the influence on mean lifespan (S3C Fig). We thus hypothesized that strong overexpression of Diedel might negatively impact normal regeneration and variably impact longevity. In support of this hypothesis, we found that levels of Diedel expression (using the TubGS driver and various doses of RU486) can positively or negatively influence longevity and that ultimately, Diedel can dose-dependently extend mean lifespan and impact age-specific mortality (Fig 3I and 3J, S3E and S3F Fig and S3 Table).

In summary, these data show that the secreted factor Diedel is a critical regulator of systemic aging. As a critical regulator of tissue communication during aging, this cytokine is both necessary and sufficient to prevent the loss of proliferative homeostasis in the aging intestine, as well as influence longevity.

### Cytokine and virokine Diedel antagonize apoptosis

**Fig 4:**
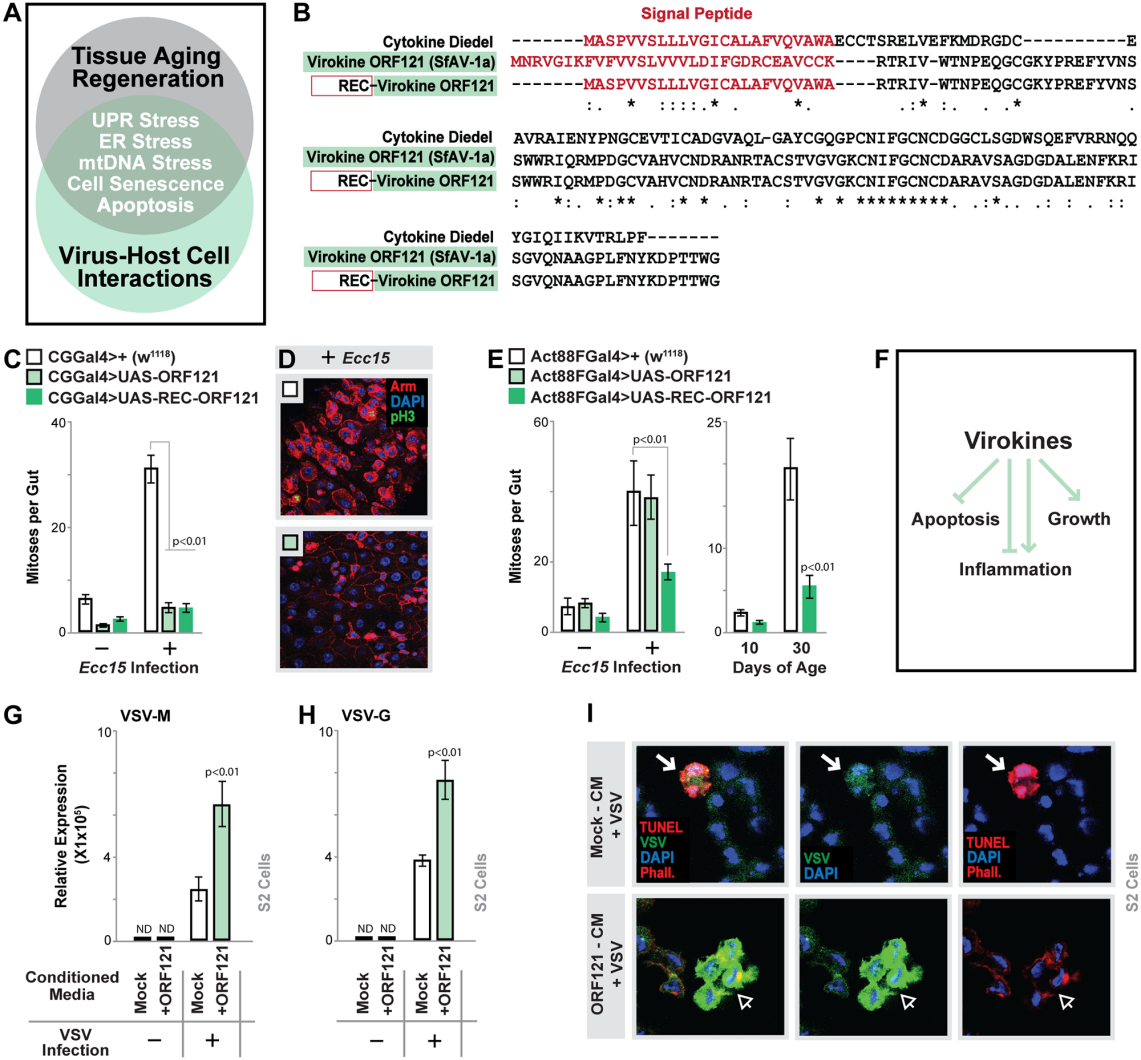
Functional analysis of Diedel viral homolog. (A) Overlap in mechanisms between tissue aging/regeneration responses and virus–host cell interactions. (B) Protein sequence alignment of *Drosophila melanogaster* Diedel, the virokine homologue (gene product ORF121) from SfAV-1a, and a recombinant ORF121 reconstituted with the *Drosophila* signal peptide (Red) and the sequence of the ORF121 mature protein. Asterisks show the homology sequence. (C) Quantification of mitoses per whole dissected midgut after *Ecc15* oral infection (Day 10, assayed by anti-pH3 immunostaining) in CGG4>UAS-ORF121, CGG4>UAS-REC-ORF121, and control (CGG4>+[w^1118^]) flies; bars represent mean ± SE, *n* = 25–30. (D) Immunostaining of dissected intestines to assess epithelial integrity of posterior midguts after *Ecc15* oral infection (Day 10), genotypes described above; pH3 (green), armadillo (“Arm”; membrane, red), and DAPI (blue). Representative images shown. (E) Quantification of mitoses per whole dissected midgut after *Ecc15* oral infection (Day 10) or during aging (assayed by anti-pH3 immunostaining) at indicated ages in CGG4>UAS-ORF121, CGG4>UAS-REC-ORF121, and control (CGG4>+[w^1118^]) flies; bars represent mean ± SE, *n* = 25–30. (F) Previously uncovered function of virokines. (G-H) Viral RNA quantification in VSV-GFP-infected *Drosophila* S2 cells (measured by qRT-PCR and normalized to *Drosophila* Rpl32), ±ORF121 conditioned media; (G) VSV-M (matrix protein) and (H) VSV-G (envelope glycoprotein); bars represent mean ± SE, *n* = 3. (I) Images of TUNEL immunostaining in VSV-GFP-infected (20 MOI) S2 cells, ±ORF121 conditioned media; TUNEL (nuclear, red), VSV-GFP (green), DAPI (blue), phalloidin (“Phall”; membrane, red); white arrow shows VSV+TUNEL+ cell (mock-conditioned media [“CM”]), and open arrow shows VSV+TUNEL− cells (ORF121 conditioned media, cells still “Phall”+[red]). Representative images shown. Underlying data can be found in S1 Data. See also S4 Fig. *Ecc15*, *E*. *carotovora carotovora 15*; ER, endoplasmic reticulum; MOI, multiplicity of infection; mtDNA, mitochondrial DNA; ND, not detectable; pH3, phospho Histone H3; qRT-PCR, quantitative real-time PCR; SfAV-1a, *S*. *frugiperda* ascovirus 1a; UPR, unfolded protein response; VSV, vesicular stomatitis virus.

We next wanted to explore potential mechanisms by which cytokine Diedel regulates systemic tissue regeneration that could can impact aging. A previous study showed that ubiquitous repression of this cytokine (using a mutant) leads to up-regulation of nuclear factor kappa B (NFkB; *Drosophila* Relish) target gene expression, highlighting that Diedel may directly influence NFkB transcriptional activation function [21]. While we observed some Diedel-dependent phenotypes associated with attenuation of NFkB function in the midgut (S5H and S5I Fig), NFkB, however, is not required for bacterial-induced regeneration [47], and presumably homeostatic clone formation as well. Thus, we hypothesized that this cytokine likely impacts age-related tissue regeneration through alternative/parallel or partially overlapping mechanisms. In order to sort through the numerous signaling pathways and stress responses that modulate regeneration in the fly intestine, which could be influenced by Diedel, we turned to Diedel homologs for guidance. Although there are no obvious homologs in vertebrate or mammalian genomes, Lamiable and colleagues previously highlighted that homologs of Diedel are found in unrelated families of DNA viruses that primarily infect lepidoptera (S6A Fig and [21, 48]). During evolution, DNA viruses have acquired an array of cellular genes from various host genomes. Termed virokines, these often-secreted proteins may act as a mimetics or antagonists of their host homologs, altering cell signaling to promote viral infection [18–20, 49]. Due to the previously mentioned overlap in host signaling mechanisms used to either (i) govern stress-related tissue regeneration or (ii) control antiviral responses (Fig 4A), we decided to explore virokine Diedel function in order to inform on the host’s gene’s role in influencing tissue degeneration during aging. First, we generated upstream activating sequence (UAS)-dependent transgenic flies capable of expressing either a full-length virokine Diedel (ORF121 from *Spodoptera frugiperda* Ascovirus 1a [SfAV-1a]) or a recombinant version in which the putative signal peptide from the viral protein was replaced with the *Drosophila* signal peptide sequence (REC-ORF121, Fig 4B). Overexpressing either ORF121 or REC-ORF121 from fat body strongly attenuated midgut regenerative responses induced by bacteria (Fig 4C and 4D), while overexpressing REC-ORF121 from muscle inhibited regenerative responses during infection and aging (Fig 4E). ORF121 expression in muscle did not impact these responses (Fig 4E), suggesting that the full-length virokine may not be readily secreted from all types of host cells. These data suggest that the viral homolog of Diedel may mimic function of the host gene and thus can inform on its function.

Virokines have been shown to influence various host responses in order to limit antiviral mechanisms and promote infection, including programmed cell death (including apoptosis), inflammation, and growth-factor signaling (Fig 4F and [50–54]). We next investigated virokine Diedel function in relation to viral infection. Utilizing a previously described in vitro infection model of *Drosophila* S2 cells with vesicular stomatitis virus (VSV, [55, 56]), we assayed the ability of secreted virokine Diedel to impact viral infection. Transfection of expression plasmids containing tagged host Diedel or REC-ORF121 in S2 cells revealed that these proteins are readily secreted into media (S6B and S6C Fig). However, as predicted from in vivo experiments (Fig 4E), tagged full-length ORF121 secretion from these cells is less efficient (ORF121-conditioned media, S4B and S4C Fig). Still, we found that conditioned media containing secreted ORF121 markedly increased the abundance of VSV-G and VSV-M RNA in infected S2 cells (monitored by qRT-PCR of viral RNA, Fig 4G and 4H). These results suggest that Diedel may increase viral replication on a per-cell basis or inhibit apoptosis, the latter of which is an important host response to viral infection to minimize both viral replication and spread. To explore a role for Diedel in limiting apoptosis, we employed an immunofluorescence approach to coordinately monitor viral abundance and TUNEL positivity. In control experiments, many VSV-GFP-positive S2 cells were observed undergoing apoptosis (monitored by TUNEL-positive nuclei, Fig 4I). However, ORF121 promoted striking increases in the amount of cellular VSV-GFP but an absence of TUNEL staining. These data suggest that secreted virokine Diedel can antagonize apoptosis to increase viral abundance and likely promote spread.

**Fig 5:**
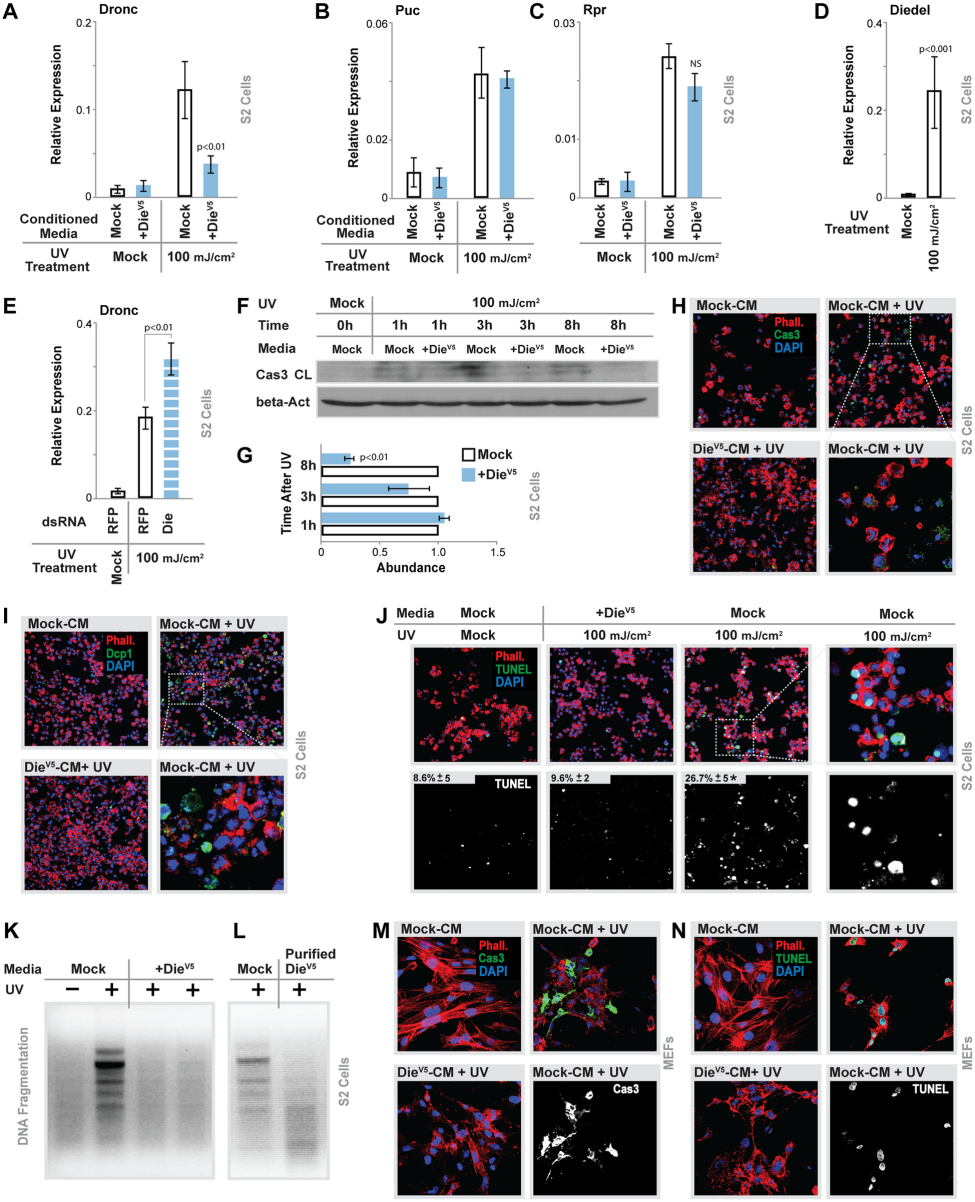
Secreted Diedel attenuates apoptosis in vitro. (A-C) *Drosophila* S2 cells exposed to UV (100 mJ/cm^2^) and treated with mock or +Die^V5^ conditioned media (A) Dronc, (B) Puc, (C) Rpr transcription (measured by qRT-PCR); bars represent mean ± SE, *n* = 4. (D) Diedel transcription (measured by qRT-PCR) in S2 cells after exposure to UV, bars represent mean ± SE, *n* = 4. (E) dsRNA targeted against RFP (control) or Diedel (“Die”) in S2 cells exposed to UV (100 mJ/cm^2^). Dronc transcription measured by qRT-PCR; bars represent mean ± SE, *n* = 3. (F-G) Immunoblot of protein extract from S2 cells exposed to UV (100 mJ/cm^2^) and treated with Die^V5^ conditioned media at time 0 h, 1 h, 3 h, and 8 h, analyzed by western blotting with cleaved Cas-3 (“Cas 3 CL”) and normalized to beta-Actin (“beta-Act”). (G) Western blot quantification (normalized to beta-Actin), bars represent mean ± SE, *n* = 3. (H-I) Images of (H) cleaved Cas-3 (“Cas 3”) or (I) cleaved Dcp-1 (“Dcp1”) immunostaining in S2 Cells exposed to UV (100 mJ/cm^2^) and treated with mock or Die^V5^ conditioned media (“CM”). (H) Cas-3 or (I) Dcp-1 (green), phalloidin (“Phall”; membrane, red), DAPI (blue). Bottom-right panel shows (5× zoom) Cas-3+ or Dcp-1+ cells in mock-conditioned media and UV-treated cells. Representative images shown. (J) Images of TUNEL immunostaining in S2 Cells exposed to UV (100 mJ/cm^2^) and treated with mock or Die^V5^ conditioned media (“media”); TUNEL (nuclear, green), DAPI (blue), phalloidin (membrane, red). Bottom panels show TUNEL+ nuclei (white) with quantification; represents mean percent of TUNEL-positive nuclei (to total cells) ± SEM, *n* = 20–30. (K-L) Genomic DNA samples were isolated from S2 cells after UV exposition and treatment with (K) mock or Die^V5^ conditioned media (2 of 3 independent samples shown) or (L) purified Die^V5^-tagged protein and separated by agarose gel electrophoresis. (M-N) Images of (M) cleaved Cas-3 (“Cas 3”) or (N) TUNEL immunostaining in MEFs exposed to UV (100 mJ/cm^2^) and treated with mock or Die^V5^ CM. (M) Cas-3 or (N) TUNEL (nuclear, green), phalloidin (actin filaments, red), DAPI (blue). Bottom-right panel shows isolated (white) Cas-3+ or TUNEL+ cells in mock-conditioned media and UV-treated cells. Representative images shown. Underlying data can be found in S1 Data. See also S4 and S6 Figs. Cas-3, caspase 3; Dcp-1, death caspase 1; +Die^V5^, Diedel-V5; Dronc, death regulator Nedd2-like caspase; dsRNA, double-stranded RNA; MEF, mouse embryonic fibroblast; NS, not significant; Puc, Puckered; qRT-PCR, quantitative real-time PCR; RFP, red fluorescent protein; Rpr, Reaper.

We thus further investigated the role of host cytokine Diedel in the regulation of apoptosis. Still utilizing S2 cells, we assayed the ability of secreted Diedel (Die^V5^-conditioned media, S6B–S6E Fig) to limit UV/DNA damage–induced apoptosis in vitro. Apoptosis is highly conserved in *Drosophila*, as various apoptotic responses are mediated by initiator caspases (death regulator Nedd2-like caspase [Dronc]; caspase 9 [Cas-9]-like) and effector caspases (DrICE and death caspase 1 [Dcp-1]; caspase 3 [Cas-3]-like), as well as a family of inhibitor of apoptosis (IAP; Diap1) antagonists (head involution defect [Hid], Reaper [Rpr], and Grim) governed by Jun-N-terminal kinase (JNK) activity [7, 13]. Dronc, Hid, Rpr, and Grim can be transcriptionally induced [57, 58]. We found that Diedel strongly attenuated UV-induced Dronc expression but not Hid, Rpr, Grim (only Rpr is shown), or Puckered (Puc, a classical JNK target gene) expression (Fig 5A–5C), at least suggesting that Diedel does not impact JNK-mediated apoptosis. Furthermore, Diedel is transcriptionally induced after UV-treatment in S2 cells, and Diedel inhibition (using double-stranded RNA [dsRNA]) significantly enhances UV-induced Dronc expression (Fig 5D and 5E). This cytokine also attenuates the temporal cleavage (activation) of Cas-3 after UV-treatment (visualized by western blots utilizing a mammalian cleaved Cas-3 antibody that is a marker for both effector caspase activity and Dronc activity in *Drosophila* [59], Fig 5F and 5G). Immunostains of S2 cells further revealed that secreted Diedel almost completely blocks the UV-induced activation/induction of effectors caspases (Cas-3 and Dcp-1, Fig 5H and 5I). We also monitored end points of apoptotic pathway activation (5 h after UV treatment) and found that Diedel can potently block DNA fragmentation assayed by TUNEL staining and gel electrophoresis (Fig 5J and 5K). Diedel purified from conditioned media has similar effects (Fig 5L). Diedel did not influence NFkB target gene expression at steady state or acutely in response to UV in S2 cells, suggesting that NFkB is not required for these apoptotic responses (S6F–S6J Fig).

Despite a lack of sequence homology, secreted Diedel is also capable of inhibiting apoptosis in mammalian cells (mouse embryonic fibroblasts [MEFs]) in response to UV treatment, attenuating Cas-3 activation and DNA fragmentation (TUNEL, Fig 5M and 5N), as well as blocking the induction of apoptosis-related inflammatory cytokines (tumor necrosis factor alpha [TNFα] and interleukin 1 beta [IL-1β], S8K Fig and [60]). Taken together, these data show that secreted Diedel can robustly antagonize apoptosis and cell death in vitro, potentially across taxonic groups.

### Diedel systemically governs apoptosis during aging

**Fig 6:**
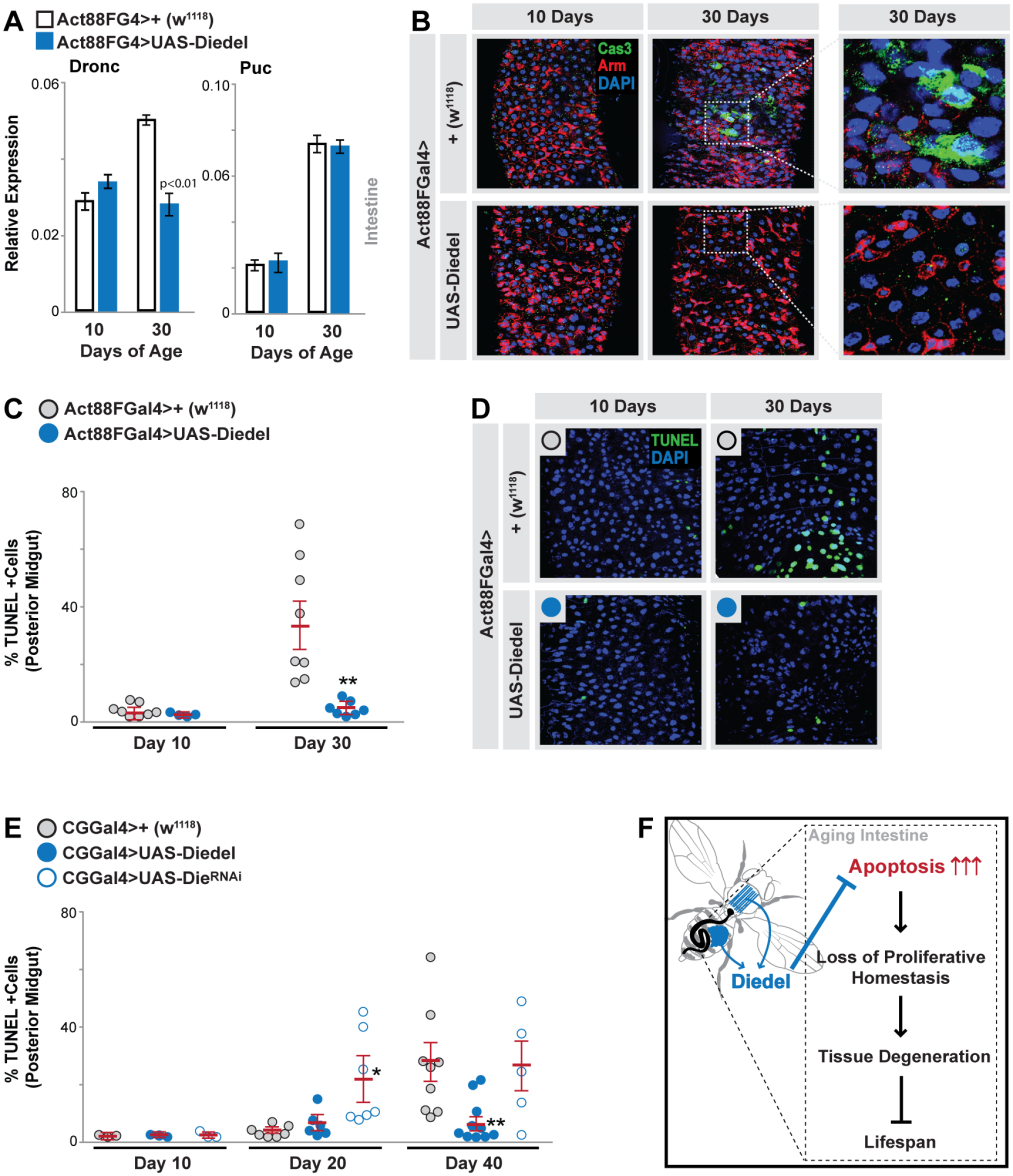
Diedel is functionally conserved and systemically governs intestinal apoptosis during aging. (A) Dronc and Puc transcription in dissected midguts (measured by qRT-PCR) from young (10 d) and old (30 d) Act88FG4>UAS-Diedel and control (Act88FG4>+[w^1118^]) flies. Bars represent mean ± SE, *n* = 4. (B) Cas-3 immunostaining (in posterior midgut) at indicated ages and genotypes; Cas-3 (green), Arm (membrane, red), DAPI (blue). Far-right panels show (5× zoom) Cas-3+ cells with enlarged nuclei (DAPI). (C-D) (C) Quantification of TUNEL+ cells (in posterior midgut) from young (10 d) and old (30 d) flies, genotypes described above. Displayed as dot plot (mean ± SE, red line), dots represent individual midguts. (D) Representative images of TUNEL immunostaining (posterior midgut) at indicated ages and genotypes; TUNEL (nuclear, green), DAPI (blue). (E) Quantification of TUNEL+ cells (in posterior midgut) from young (10 d) and old (30 d) CGG4>UAS-Diedel, CGG4>UAS-Die^RNAi^, and control (CGG4>+[w^1118^]) flies. Displayed as dot plot (mean ± SE, red line), dots represent individual midguts. (F) Proposed model depicting the role of Diedel in regulating systemic (intestinal) tissue aging and longevity. Underlying data can be found in S1 Data. See also S5 and S6 Figs. Arm, armadillo; Cas-3, caspase 3; Dronc, death regulator Nedd2-like caspase; Puc, Puckered; qRT-PCR, quantitative real-time PCR.

Apoptosis is also required for normal tissue regeneration, but misregulation of apoptotic responses is increasingly linked to tissue aging [61, 62]. Apoptosis has thus emerged as a critical antagonistic response to primary causes of cellular damage during aging and must be precisely modulated to ensure proper tissue architecture and epithelial function. In the *Drosophila* intestine, apoptotic responses in enterocytes are required for both homeostatic regeneration/tissue growth and pathogen-induced regeneration through the production of mitogenic signals [63–66]. These paracrine signals drive stem cell proliferation and differentiation. Critically, overactivation of apoptotic responses in enterocytes promotes hyperproliferation and tissue degeneration [63]. Thus, we next wanted to determine if secreted Diedel could influence apoptosis in vivo in the aging midgut. Similar to our in vitro findings, Diedel expression in muscle (Act88FGal4>UAS-Diedel) inhibits age-related increases in intestinal Dronc expression but not JNK-target genes (Fig 6A). We also found a marked increase in apoptosis and cell death in the aging midgut (assayed by TUNEL staining and Cas-3 immunostaining), and muscle-specific Diedel expression significantly blocks these age-related increase in apoptosis (Fig 6B–6D and S7A–S7C Fig), which correlates with an attenuation of ISC hyperproliferation and tissue dysplasia (Fig 2E). Alternatively, Diedel knock-down in fat body (using CGGal4 and PplGal4 coupled with RNAi) leads to hastened induction of apoptosis and cell death in the aging midgut (Fig 6E and S7D–S7H Fig), which correlates with accelerated aging of the intestine (Fig 3E). Furthermore, secreted Diedel can inhibit cytokine (unpaired 3 [Upd3])-mediated Janus kinase (Jak)/signal transducer and activator of transcription proteins (Stat) signaling, which in turn regulates stress- and age-related stem cell proliferation/midgut regeneration (reviewed in [67]). Both in vitro and in vivo, Diedel indirectly attenuates Jak/Stat activation by repressing stress-induced Upd3 transcriptional up-regulation (S8A–S8J Fig). We also confirmed that apoptotic responses can drive Upd3 induction and Jak/Stat activation (S6E Fig). In summary, these data show that cytokine Diedel can systemically modulate apoptosis, and subsequently control regenerative signals, during tissue aging.

Combined with our other findings, our data suggest that Diedel governs apoptosis to ultimately control proliferative homeostasis, tissue degeneration, and aging (Fig 6F).

## Discussion

Overall, our results reveal that primary causes of cellular aging can have tissue-autonomous and systemic effects on integrative responses that drive age-related phenotypes, highlighting a systemic coordination in tissue aging. Furthermore, we uncovered a systemic signaling mechanism (mediated by the cytokine Diedel) that is capable of promoting communication between muscle/adipose and the regenerating midgut during aging in *Drosophila*. This cytokine is both necessary and sufficient to limit age-related tissue degeneration and extend lifespan, and our data highlight Diedel as a critical regulator of aging and longevity.

Diedel appears to impact tissue aging through the attenuation of apoptosis. Apoptosis is the major programmed cell death pathway used to remove unneeded or harmful cells during development or tissue maintenance/regeneration. Increasing evidence also suggests that apoptosis may be a driving force of tissue aging, albeit extremely pleiotropic. Too little can promote cancer or age-related (and harmful) cell senescence, while too much can promote tissue atrophy or hyperplastic overgrowth through compensatory proliferation [61, 62]. The loss of proper apoptotic responses during aging is thus likely to be influenced by changes in both pro- and antiapoptotic mechanisms. An abundance of precisely regulated mammalian cytokines act as critical proapoptotic signals to control various programmed cell death pathways, especially those related to immune cell regulation (reviewed in [60]). Diedel emerges as an intriguing antagonist of these responses, as it is a secreted factor under tight transcriptional control (similar to other stress response genes and cytokines), allowing it to function as a systemic rheostat to properly balance apoptosis.

While *Drosophila* Diedel is able to attenuate activity of the apoptotic pathway in both fly and mammalian cells, the underlying mechanism remains unclear. Apoptosis is governed by both intrinsic and extrinsic pathways. Extrinsic receptor-induced signaling (triggered at the cell surface by ligands such as TNFα and IL-1β) and the release of intrinsic apoptogenic factors (triggered by changes in the permeabilization of mitochondrial membranes) ultimately converge on activation of downstream caspases [68–70]. JNK, through the induction of mitochondrial-localized IAP antagonists, plays a crucial role in the intrinsic activation of apoptosis, especially in *Drosophila* [7, 58, 71]. Our data suggest that secreted Diedel does not influence JNK activation after cell damage, implying that this cytokine attenuates apoptosis through an extrinsic pathway and putative unknown receptor. Diedel’s potentially unique role in regulating apoptosis may influence other signaling pathways as well, such as immune/NFkB responses [21], known to share common control elements [72]. Furthermore, Diedel may also directly influence NFkB in certain cell types (39) and thus impact apoptosis through governing NFkB activity. A more detailed spatiotemporal analysis of Diedel molecular function is required to understand how various signaling pathways intersect to control outputs. The exact fate of Diedel-treated cells also needs further examination, as it remains unclear if these cells are just temporally blocked from cell death or display more permanent features that could be beneficial, detrimental, or likely both. Exploring this protein’s unique function is likely to inform on how secreted antagonists of apoptosis precisely modulate cell death in concert with extrinsic and intrinsic activators. Furthermore, uncovering the underlying mechanism by which Diedel influences apoptotic responses will also shed light on the complex spatiotemporal role of programmed cell death pathways in aging phenotypes.

Our data show that Diedel expression increases with age in *Drosophila* and that the cytokine limits age-related induction of apoptosis and concurrent loss of proliferative homeostasis in the aging intestine. However, Diedel can be induced in response to various stressors, suggesting a broad role in regulating apoptosis beyond just that associated with midgut regeneration. Our data also imply that Diedel, and antiapoptotic mechanisms in general, may be critical for beneficial hormetic responses. Age-related hormetic effects induced by various antagonistic stress responses appear to have diverse systemic components, which are likely to exert broad effects on cellular targets [32, 37, 73–75]. Because change in the regulation of apoptosis is a general feature of many aging metazoans, Diedel may represent an important secreted factor that systemically communicates various types of cell damage/stress between tissues during aging, generally eliciting beneficial effects.

Diedel’s potentially broad role in regulating apoptosis is perhaps best highlighted by its viral counterparts. Diedel, like other host genes from various species—including humans—has been hijacked by viral genomes. During evolution, DNA viruses have acquired a broad array of genes from hosts in order to shift virus–host cell interactions toward survival of virus-infected cells [18, 19, 21, 53, 76, 77]. Encoded virokines often act as mimetics or antagonists of their cellular homologs, and we find that virokine Diedel can also limit apoptosis during viral infection, as well as attenuate age-related tissue degeneration in vivo. These data show that viral genomes may contain vital information associated with underlying mechanisms that govern aging and can perhaps be exploited to uncover novel modulators and potential therapeutics related to tissue aging. Using Diedel as an example, secreted virokines have been proposed as putative immunomodulatory therapeutics [19, 78], and there is a long history of targeting cell death (apoptosis) pathways for drug development related to cancer and other age-associated diseases [79, 80]. Finally, while most virus-acquired host genes are linked to evolution of pathogenic virus–host cell interactions, mutualistic relationships also exist in which viruses are beneficial to their hosts [81, 82]. Thus, secreted viral proteins emerging from symbiotic or “good” viruses might also have the capability to influence host-tissue homeostasis during stress or aging, similar to mutualistic microorganisms.

## Materials and methods

### *Drosophila* stocks and culture

The following strains were obtained from the Bloomington *Drosophila* Stock Center: w1118, Act88FGal4 (38461). UAS-ERCC1RNAi (110419) was obtained from the Vienna Drosophila RNAi Center. UAS-MEI9RNAi was a gift from J. Sekelsky, CGGal4 was a gift from C. Thummel, PplGal4 was a gift from M. Pankratz, TubGeneSwitch was a gift from H. Jasper, NP1Gal4 was a gift from D. Ferrandon, and 10xSTAT-GFP was a gift from E. Bach. UAS-DiedelRNAi transgenic lines were generated with plasmid (dna571) from the Vienna *Drosophila* RNAi Center. UAS-Diedel, UAS-ORF121, UAS-REC-ORF121, Diedelp-RFP, Act88FGeneSwitch, and UAS-DiedelRNAi transgenic flies were generated for this study. NOTE: All flies used in this study were backcrossed ×10 into the w1118 background, with continued backcrossing every 6–8 mo to maintain isogenecity.

All flies were reared on standard yeast- and cornmeal-based diet at 25 °C and 65% humidity on a 12-h light/dark cycle, unless otherwise indicated. The standard lab diet (cornmeal-based) was made with the following protocol: 14 g Agar/ 165.4 g Malt Extract/ 41.4 g Dry yeast/ 78.2 g Cornmeal/ 4.7 ml propionic acid/ 3 g Methyl 4-Hydroxybenzoate/ 1.5 L water. For RU486 food, RU486 or vehicle (ethanol 80%) was mixed with food, resulting in a 200 uM concentration of RU486 in the food, unless otherwise indicated.

All analysis was exclusively done in female flies (with the exception of lifespan assays) because of sex-specific differences in midgut regeneration.

### Generation of transgenic *Drosophila*

UAS-Diedel flies were generated by PCR amplification of adult Drosophila (w1118) cDNA, with specific primers, and then cloned into the pUASt plasmid. DiedelP-RFP flies were generated by PCR amplification of Diedel promoter sequence with specific primers from w1118 genomic DNA and clone in pB-RFP plasmid [83]. Act88FGeneSwitch flies were generated by PCR amplification of Act88F promoter (from w1118 flies, including TATAbox) and cloned into pP(wlo+hsinGs) plasmid without the hsp minimal promoter [84]. UAS-DiedelRNAi transgenic lines were generated with plasmid (dna571) from the Vienna *Drosophila* RNAi Center (in the pUASt plasmid).

UAS-ORF121 and UAS-RED-ORF121 were generated by custom nucleotide synthesis of ORF121 gene from SfAV-1a and REC-ORF121 (*Drosophila* signal peptide with ORF121 mature protein sequence) provided by gBlock:IDT, and subsequently cloned into the pUASt plasmid. Sequences are as follows:

ORF121—from SfAV-1a:

taatatgcggccgcATGAACCGAGTTGGAATTAAGTTTGTGTTTGTTGTTTCGCTGGTTGTTGTACTCGATATATTCGGTGATCGATGTGAAGCGGTGTGTTGTAAACGAACACGTATCGTGTGGACGAATCCGGAACAGGGTTGTGGAAAGTATCCGAGAGAATTCTACGTAAATTCCAGCTGGTGGAGGATTCAACGCATGCCCGACGGTTGTGTGGCGCACGTTTGTAACGACCGGGCGAATCGCACGGCATGTTCTACTGTGGGCGTTGGCAAGTGTAACATTTTCGGGTGCAACTGTGACGCGCGGGCTGTATCGGCGGGTGATGGAGACGCATTGGAGAATTTCAAGAGAATCAGCGGCGTACAGAATGCCGCTGGCCCTCTCTTCAATTACAAAGATCCCACCACTTGGGGTTGActcgagtaatat

REC-ORF121 sequence:

taatatgcggccgcATGGCATCCCCAGTAGTCAGCCTGCTTCTCGTCGGGATCTGCGCCCTGGCCTTCGTCCAGGTGGCCTGGGCGCGAACACGTATCGTGTGGACGAATCCGGAACAGGGTTGTGGAAAGTATCCGAGAGAATTCTACGTAAATTCCAGCTGGTGGAGGATTCAACGCATGCCCGACGGTTGTGTGGCGCACGTTTGTAACGACCGGGCGAATCGCACGGCATGTTCTACTGTGGGCGTTGGCAAGTGTAACATTTTCGGGTGCAACTGTGACGCGCGGGCTGTATCGGCGGGTGATGGAGACGCATTGGAGAATTTCAAGAGAATCAGCGGCGTACAGAATGCCGCTGGCCCTCTCTTCAATTACAAAGATCCCACCACTTGGGGTTGActcgagtaatat

pUAS-Diedel, pUAS-ORF121, pUAS-REC-ORF121, pP(ACT88FGS), and pUAS-DiedelRNAi plasmids were injected into w1118 embryos, and pB-DiedelP-RFP was injected into w1118; attp40 embryos with a phiC31 integrase helper plasmid (Rainbow Transgenic Flies).

### In vivo immunostaining and microscopy

For intestines, intact midguts (at indicated ages) were dissected and fixed at room temperature for 20 min in 100 mM glutamic acid, 25 mM KCl, 20 mM MgSO4, 4 mM sodium phosphate, 1 mM MgCl2, and 4% formaldehyde. All subsequent incubations were done in PBS, 0.5% BSA, and 0.1% Triton X-100 at 4 °C. The following primary antibodies were used: mouse anti phospho-Histone 3 (cell signaling 9701, 1:1,000), cleaved Cas-3 (cell signaling 9661, 1:1,000), cleaved Dcp-1 (cell signaling 9578, 1:1,000), mouse anti-armadillo N2 7A1 (1:100), and mouse anti-LacZ (40-1a) (1/500) from Developmental Studies Hybridoma Bank. Fluorescent secondary antibodies were obtained from Jackson Immunoresearch. Hoechst (1:1,000) was used to stain DNA.

For muscle immunostaining, longitudinal thoracic muscle segments (at indicated ages) were dissected in PBS and fixed with 4% paraformaldehyde for 20 min at room temperature, washed 3 times with PBS containing 0.1% Triton X-100 (PBST), and then blocked in blocking buffer (5% BSA in PBST) for 1 h. Primary antibodies (Anti-Ubiquitinine from Enzo [BML-PW8805-0500, 1:500] and Anti- H2aV From Rockland [600-401-914, 1:500]) were applied overnight at 4 °C. Alexa Fluor-conjugated secondary (Jackson Immunoresearch, 1:500) antibodies were incubated for 2 h at room temperature. Hoechst was used to stain DNA, and Phalloidin (FITC or Rhodamin from ThermoFisher [F432, R415]) was used to stain F-Actin fibers.

Confocal images were collected using a Nikon Eclipse Ti confocal system (utilizing a single focal plane) and processed using the Nikon software and Adobe Photoshop.

### Lifespan analysis

Thirty virgins were crossed to 10–15 males of the desired genotype for all survival experiments unless otherwise indicated (see below). After initial mating, parental flies were allowed 2 d to lay eggs before being removed from bottles. Progeny of crosses was collected for 3–4 d after initial eclosion. Collected progeny were allowed to mate for 2 d at room temperature. Progeny were then separated according to sex and genotype into cages (around 75–100 flies/cage) and aged at 25 °C with constant humidity (approximately 65%).

In order to normalize population densities for RU486 dose-dependency longevity experiments (Fig 3I and 3J), 75 virgin flies were mated with 50 males and allowed to lay eggs for less than 24 h on apple plates, supplemented with live yeast paste. Eggs were collected from the apple plate by washing in 1X PBS solution into 15 ml tubes. Then, 18–20 μL of egg suspension was dispensed into 200-mL bottles containing standard lab food. Two days after eclosion, once-mated flies were transferred to plastic cages at a density of 100–120 flies per cage.

For all independent populations, plastic cages (175-ml volume, 5-cm diameter from Greiner Bio-One) were used for lifespan experiments. Food, changed every 2–3 d, was provided in vials inserted into a foam plug (4.9 cm in diameter, 3-cm thick from Greiner Bio-One). For RU486-dependent experiments, RU486 or vehicle (ethanol, 80%) was mixed with food, resulting in a 200 μM concentration of RU486 in the food (unless otherwise indicated for dose response experiments). Dead flies were counted every 2–3 d. Survival data were analyzed using Prism statistical software.

All independent lifespan analyses for independent trials are included in the S2 Data.

### Mortality estimation

Mortality rate was calculated as described in [85]. N0 is the number of individuals in the initial cohort, as well as Nx, the number alive at the start of each day. The probability of surviving from age x to age x + 1, given the individual is alive at the start of age x, is Px = Nx + 1/Nx. The age-specific rate of mortality is estimated as μx = −ln(Px). The mortality rate is plotted as ln(μx).

### ISC lineage analysis

The previously developed marked lineage system was used to generate clones of lacZ-expressing cells [86]. In this system, flippase-mediated recombination fuses the α-tubulin promoter to the lacZ gene, allowing transcription of lacZ. In the absence of heat shock, X15 flies carry 2 inactive tubulin-promoter lacZ (X-15–29 and X-15–33). Upon heat shock induction of flippase in dividing cells, these transgenes can recombine through the flippase recombination target (FRT) region, generating an active lacZ transgene. Thereafter, cells carrying the recombined lacZ transgene, as well as its progeny, will be marked by constitutive production of ß-galactosidase. The size of the marked clone is a direct measure of the division rate. To induce somatic recombination, adult flies were heat-shocked at 10 d or 30 d of age at 37 °C for 60 min. Midguts were dissected 5 d after heat shock and stained with anti-LacZ antibody.

### In vivo TUNEL (cell death) assay

Intact midguts were dissected (at indicated ages) in 1× PBS and fixed in 4% paraformaldehyde for 25 min at room temperature. Samples were washed with 1× PBT for 5 min. Cell death was detected by using In Situ Cell Death Detection Kit (Roche) according to manufacturer’s instruction.

### qRT-PCR analysis

Total RNA from dissected intact fly thorax, carcass (all of the eggs and intact intestines were removed) and midguts, or S2 cells and MEFs, were extracted using Trizol and complementary DNA synthesized using Superscript II (Invitrogen). RT-PCR was performed using SYBR Green (biorad), the Applied Biosystems StepOnePlus Real-Time PCR systems, and the primer pairs described in the extended experimental procedures (see S4 Table). Results are average ± standard error of at least 3 independent samples, and quantification of gene expression levels calculated using the ΔCt method and normalized to actin5C for all in vivo experiments or rpl32 for all S2 cell in vitro experiments (virus and UV-treatment) or GAPDH for all MEF in vitro experiments. The standard curve method was used to quantify dose-dependent Diedel mRNA.

### Enteropathic infection assay

Ecc15 was used for natural (oral) infections. Briefly, for oral infection, flies (10 d of age) were placed in a fly vial with food/bacteria solution and maintained at 25 °C. The food solution was obtained by mixing a pellet of an overnight culture of bacteria (OD 200) with a solution of 5% sucrose (50/50) and added to a filter disk that completely covered the surface of standard fly medium. Midguts of infected flies were dissected 16 h after oral contact with infected food. At least 3 vials (cohorts of 15 flies per vial) were used for each genotype for subsequent analysis (immunostaining and qRT-PCR)

### RNA-seq analysis

Intact fly thorax and midguts were dissected (at indicated ages) in PBS. Total RNA was extracted using Trizol reagent and used as template to generate sample libraries for RNA sequencing (using the TruSeq Stranded Total RNA Library Prep Kit). Sample libraries were sequenced using the Illumina HiSeq 2500. Sequence cluster identification, quality prefiltering, base calling, and uncertainty assessment were done in real time using Illumina’s HCS 2.2.58 and RTA 1.18.64 software with default parameter settings. Between 7 and 12 million (1X50) base pair reads were generated per library and mapped to the *Drosophila* genome (Release 6). Expression was recorded as reads per kbp per million reads (RPKM). GO clustering analysis was performed using FlyMine. FASTQ data files representing unique libraries are deposited in the NCBI Gene Expression Omnibus database (GSE111248).

### Feeding behavior assay

The CAFE assay was performed as follows: Briefly, a single fly was transferred from standard food to vials filled with 5 mL of 1.5% agar that maintains internal humidity and serves as a water source. Flies were fed with 5% sucrose solution maintained in 5-μl capillaries (VWR, #53432–706). After 12 h of habituation, the old capillaries were replaced with a new one at the start of the assay. The amount of liquid food consumed was recorded after 24 h and corrected on the basis of the evaporation (typically <10% of ingested volumes) observed the identical vials without flies. Five flies were weighed in order to normalize samples.

### Climbing assay

Twenty flies (at indicated ages) were placed into the empty vials, taped to the bottom, and then given 30 s to climb a distance of 6 cm. Flies that successfully climbed 6 cm or beyond in 30 s were counted. At least 100 total flies (5 cohorts) were used for each genotype tested.

### Commensal quantification

To culture commensal bacteria, dissected midguts were homogenized in 100 μl PBS using pestles, and different dilutions of this suspension were plated onto mannitol agar plates. Plates were incubated at 30 °C for 36–48 h, and the number of colonies per plate was counted.

### Cell culture conditions and UV treatment

*Drosophila* S2 cells (obtained from *Drosophila* Genomics Resource Center) were maintained in Schneider’s *Drosophila* media supplemented with 10% FCS, 50 U/ml penicillin, and 50 μg/ml streptomycin at 25 °C. Primary MEFs were cultured in DMEM supplemented with 10% FCS, 50 U/ml penicillin, and 50 μg/ml streptomycin at 37 °C in 5% CO2. To induce apoptosis, the cell media was removed, and cells were treated with ultraviolet C light (UVC; dose as indicated).

### In vitro transfection and associated cloning

Diedel, Upd1, and Upd3 were amplified from cDNA of w1118 flies using specific primers (see Primer Table) and cloned into pAC5.1 V5-His plasmid. Upd2 was amplified from Upd2 cDNA obtained from DGRC (1647385) and then cloned in the pAC5.1 V5-His plasmid. ORF121 gene (from SfAV-1a) sequence and Rec-ORF121 sequence were generated by nucleotide synthesis (gBlock:IDT), amplified with specific primers (see S4 Table), and cloned into the pAC5.1 V5-His plasmid. After sequencing, 1 μg of plasmids was transfected into S2 cells (2.106) using Effecten transfection Kit (Qiagen).

### In vitro dsRNA treatment

Regions of cDNA for Diedel, Diap-1, and RFP (control) were amplified by PCR from plasmids containing cDNA sequence (Berkeley *Drosophila* Genome Project) or appropriate plasmids. Each primer used in the PCR contained a T7 polymerase binding site (GAATTAATACGACTCACTATAGGG AGA) at the 5′ and 3′, followed by sequences specific for: Diedel (forward: CGCCCAGCGAAAAATGTATTGATTGATGGC, reverse: CCGAATGCTCTCAGCAATAATGCTTAGTCT), Diap-1 (forward: GCAACAGGGCATCCTAACC, reverse: TCGCAAAACTCCAC CTTCTC), and RFP (forward: CCGTAAGAATGTAGCCAAAATG, reverse: GTCGCTGAAACGCAGAATG). The PCR products were gel purified, and complementary RNA strands were synthesized by in vitro transcription using the T7 Megascript Kit (Ambion). To obtain dsRNA, sense and antisense strands were annealed by heating to 65 °C for 15 min and then allowed to cool to room temperature. The quality of the dsRNA was analyzed on an agarose gel and stored at −80 °C. For knock-down experiments, S2 cells were seeded in 6-well plates in 2 ml of medium the day before the RNAi procedure. The following day, medium was removed, and dsRNA (10 μg) was directly added to 1 ml of serum-free media and mixed vigorously. Cells were incubated for 1 h, followed by the addition of 2 ml of media supplemented with 10% FBS. The S2 cells were incubated for 2 d to induce posttranscriptional gene silencing.

### Western blot analysis

S2 Cells were harvested at various times after UV treatment, washed in ice-cold PBS, and incubated on ice for 30 min in lysis buffer (50 mM Tris-HCl, pH 8.0, 150 mM NaCl, 1% triton), 100 μM PMSF, and complete protease inhibitor. Cell lysate was centrifuged at 14,000 g for 20 min at 4 °C, and the amount of protein in the supernatant was quantified by Bradford protein assay (Bio-Rad Laboratories). An aliquot of protein from each sample was boiled for 10 min in Laemmli buffer before loading onto an SDS polyacrylamide gel. Proteins were transferred onto nitro-cellulose membranes and blocked for 1 h in TBST (50 mM Tris-HCl, pH 7.5, 150 mM NaCl, 0.05% Tween 20) containing 5% BSA. Protein immunoblots were performed using antibodies against cleaved Cas-3 (cell signaling, #9661), V5 tag (Sigma, V8137), pH2AvD (UNC93-5.2.1 was deposited to the DSHB by Hawley, R.S. [DSHB Hybridoma Product UNC93-5.2.1]), and beta-Actin (Cell signaling, #4970). The immobilized proteins were further incubated with the corresponding HRP-conjugated secondary antibody, and protein bands were visualized using a commercial chemiluminescence detection kit (ECL,Amersham).

### Viral infection assay

VSV-GFP [87] was propagated and assayed on BHK cells. In brief, confluent BHK cells were infected with VSV and incubated with virus until complete infection occurred (1 d). Cells were centrifuged at 2,000 rpm for 5 min, and supernatant was collected. Virus was concentrated by centrifuging at 25,000 g for 1.5 h at 4 °C. The pellet was resuspended in sterile PBS and stored at 80 °C. A standard plaque assay on BHK cells was performed on all purified virus stocks before use. S2 cell infections were performed in serum-free Schneider’s media for 1 h using a multiplicity of infection (MOI) of 20 (as previously described, [55]); then, conditioned media containing serum was added for the duration of the experiment. Sixteen hours after infection, cells were processed for either RNA collection (and subsequent qRT-PCR, see S4 Table) or immunostaining (TUNEL assay).

### In vitro TUNEL (cell death) assay

S2 cells or MEFS were fixed on a poly-L-lysine (VWR)-coated glass coverslip with 10% formaldehyde for 10 min and washed 2 times with PBS. Cell death was detected by using an In Situ Cell Death Detection Kit (Roche) according to manufacturer’s protocol. Following washes in PBS, cells were stained with Alexa Fluor FITC phalloidin (Life Technologies) in PBST with Hoechst for 30 min at room temperature.

### In vitro DNA fragmentation analysis

DNA ladder fragmentation was detected as described [88], with some modifications. Briefly, S2 cells (2 × 106) were seeded into 6-well plates. The next day, the cell media was removed, and cells were exposed to UV using Stratlinker1800. The cells were treated for 5 h with conditioned media with or without Die^V5^. At the end of incubation, the DNA was extracted from S2 cells with 2 phenol/chloroform extractions and 1 chloroform extraction and finally precipitated with NaCL (300 Mm) and ethanol. The DNA was incubated for 1 h at 37 °C with RNAse A. The fragmented DNA was electrophoresed on 2% agarose gel.

### Diedel purification

The Diedel-V5-His recombinant protein was overexpressed in S2 cells at a cell density of 3.106 cells ml^−1^. After 2 d, insoluble material was removed from the harvested medium by centrifugation at 4 °C/4,000 g for 5 min. Diedel-V5-His was purified using HisPur Ni-NTA Spin Columns, 3 mL (#88226, Thermofisher), according to manufacturer’s protocol. Briefly, Diedel-V5-His supernatant was equilibrated in PBS with 20 mM Imadazole. After the supernatant was loaded onto the Ni-NTA Spin columns, tagged Diedel was eluted with equilibration buffer containing 200 mM imidazole. The tagged Diedel fractions were identified by western blot, followed by Coomassie blue staining.

## Supporting information

S1 Fig Characterization of transgenic flies. **Related to** Fig 1. (A-D) Mei-9 and ERCC1 heterodimers constitute a conserved DNA-repair endonuclease involved in NER pathway. (B) Silencing Mei-9 (UAS-Mei-9 RNAi) or Mei-9 and ERRC1 concurrently (UAS-Mei-9 RNAi, UAS-ERCC1 RNAi) in the larva epidermis (A58Gal4) inhibits DNA repair after UV irradiation. Images of adult abdominal cuticle after larval UV treatment. (C) Immunoblot of protein extract from dissected thorax of Act88FG4>+(w^1118^) controls (Day 10) and Act88FG4>UAS-Mei-9 RNAi (Day 10) flies analyzed by western blotting with pH2AvD (normalized to beta-Actin [“beta-Act”]). (D) Western blot quantification; bars represent mean ± SE, *n* = 3. (E-L) Characterization of Act88FGeneSwitch driver specificity (utilizing UAS-nlsGFP). Act88FGS>UAS-GFP flies were fed for 5 d with mock-treated food (80% ethanol control) or RU486-treated food (200 uM) to induce GFP expression. Act88FGS>UAS-GFP flies display RU486-dependent GFP expression specifically in the thoracic skeletal muscle—including the longitudinal IFMs, dorsal lateral muscles, dorsal ventral muscles—and weaker expression in leg muscles. No GFP expression is observed in any tissue in the carcass or head. Representative images of (E) whole female (F) or male flies’ (G) dissected thorax; (H) head; (I) abdomen; (J) intestine; (K) ovaries; and (L) testes. Underlying data can be found in S1 Data. GFP, green fluorescent protein; IFM, indirect flight muscle; NER, nucleotide excision repair; pH2AvD, phospo-Histone 2A gamma; RNAi, RNA interference; RU486, mifepristone.
(TIF)

S2 Fig Mortality associated with Act88FGal4 and CGGal4 survival experiments. **Related to** Figs 1, 2 and 3. (A-B) Mortality plots (female flies) associated with mu-specific (A) inhibition of Mei-9 (UAS-Mei-9 RNAi) using the Act88FGal4 driver (compared to Act88FG4>+[w^1118^] controls) and (B) overexpression of Diedel using the Act88FGal4 driver (compare to Act88FG4>+[w^1118^] controls). (C) Mortality plots (female flies) associated with fat body specific inhibition of Diedel (UAS-Die RNAi using the CGGal4 driver, compared with CGG4>+[w^1118^] controls). These plots correspond to survival analysis found in Figs 1C, 2G and 3G respectively. Underlying data can be found in S1 Data. mu-specific, muscle-specific; RNAi, RNA interference.
(TIF)

S3 Fig Mortality associated with GeneSwitch survival experiments. **Related to** Figs 1–3. (A-C) Mortality plots (female flies) associated with the mu-specific GeneSwitch inducible driver (Act88FGS) (A) Act88GS>+(w^1118^) +RU486 compared with −RU486 (vehicle alone) sibling controls; (B) Act88FGS>UAS-Mei-9^RNAi^ +RU486 compared with −RU486 (vehicle alone) sibling controls; (C) Act88FGS>UAS-Diedel +RU486 compared with −RU486 (vehicle alone) sibling controls. These plots correspond to survival analyses found in S2 Table, Figs 1D and 2H, respectively. (D-F) Mortality plots (female flies) associated with ubiquitous GeneSwitch inducible driver (Tubulin(“Tub”)GS, +RU486 compared with −RU486 [vehicle alone] sibling controls). (D) TubGS>UAS-Die^RNAi^. (E) TubGS>+(w^1118^). (F) RU486 dose dependency of mortality in TubGS>Diedel female flies, utilizing 20 mM, 50 mM, and 100 mM doses of RU486. These plots correspond to survival analyses found in Fig 3H, S2 Table, and Fig 3J/S2 Table, respectively. Underlying data can be found in S1 Data. mu-specific, muscle-specific; RU486, mifepristone.
(TIF)

S4 Fig Tissue specificity and dose dependency of DNA repair attenuation. **Related to** Figs 1 and 2. (A-B) Knock-down of Mei-9 (UAS-Mei-9 RNAi) specifically in the adult thoracic muscle (Act88FGal4) has no effect on (A) feeding behavior (measured by CAFE assay, bars represent mean ± SE, *n* = 4 independent samples) or (B) climbing (bars represent mean ± SE, *n* = 5 cohorts of 20 flies) compared with Act88FG4>+(w^1118^) controls. (C-D) Knock-down of Mei-9 and ERRC1 concurrently (UAS-Mei-9 RNAi, UAS-ERCC1 RNAi) specifically in thoracic muscle has no effect on (C) lifespan (survival curves, S2 Table) or (D) age-related intestinal stem cell hyperproliferation (quantified by pH3-positive cells [mitoses per gut] at 30 d, bar represents mean ± SE, *n* = 25–30) compared to Act88FG4>+(w^1118^) controls. (E-F) Knock-down of Mei-9 (UAS-Mei-9 RNAi) specifically in intestinal enterocytes (NP1Gal4) leads to tissue-autonomous (E) accumulation of DNA damage (measured by immunostaining with pH2AvD antibody in dissected midguts), (F) increases in intestinal stem cell mitoses (quantified by pH3-positive cells at day 10, 20, and 30; bars represent mean ± SE, *n* = 25–30), and (G) decreases in lifespan (survival curves, S1 Table) compared with NP1G4>+(w^1118^) controls. (H-I) (H) RPKM values for select, basally high genes that show no change upon muscle-specific depletion of MEI-9 in all of the unique tissue transcriptomes (thorax/muscle and intestine). Plotted on graph (I); log scale; each line represents a unique gene. (J-K) Intestinal immune gene induction during aging in Act88FG4>UAS-Mei-9^RNAi^ flies, (J) Diptericin (“Dpt”) and (K) Drosomycin (“Drs”) measured in dissected midguts from young (10 d) and old (30 d) flies by qRT-PCR; bars represent mean ± SE, *n* = 3; compared with Act88FG4>+(w^1118^) controls. Underlying data can be found in S1 Data. pH2AvD, phospho-Histone 2A gamma; pH3, phospho-Histone H3; qRT-PCR, quantitative real-time PCR; RNAi, RNA interference; RPKM, reads per kbp per million reads.
(TIF)

S5 Fig Systemic Diedel is critical to maintain intestinal homeostasis. **Related to** Figs 2 and 3. (A) Diedel transcription (measured by qRT-PCR) in Act88fG4>UAS-Diedel whole flies; bars represent mean ± SE, *n* = 3; compared with Act88FG4>+(w^1118^) controls. (B) Diedel knock-down (UAS-Die RNAi) specifically in the thoracic muscle (Act88FGal4) has no effect on intestinal stem cell proliferation during aging (measured by number of pH3-positive cells at day 10, 25, and 30; bars represent mean ± SE; *n* = 25–30) compared to Act88FG4>+(w^1118^) controls. (C) UAS-Diedel RNAi efficiency was evaluated in the fat body after sterile wounding. Diedel is induced after sterile wounding in the control flies (CGG4>+), and this induction is inhibited in CGG4>UAS-Die^RNAi^ animals; bars represent mean ± SE; *n* = 3; whole flies were used in this analysis. (D) Knock-down of Mei-9 (UAS-Mei-9 RNAi) specifically in thoracic muscle (Act88FGal4) induces accelerated and age-related activation of a Diedel reporter; displayed by an increase in the proportion of Diedel-RFP-positive flies (Diedel^P^-RFP +) in Act88FG4>UAS-MEI9^RNAi^ animals compared with Act88FG4>+(w^1118^) controls. (E) Knock-down of Mei-9 (UAS-Mei-9 RNAi) specifically in thoracic muscle (Act88FGal4) promotes elevated Diedel transcription in the carcass/fat body during aging (measured by qRT-PCR of dissected carcass in young [10 d] and old [30 d] flies; bars represent mean ± SE, *n* = 3) compared with Act88FG4>+(w^1118^) controls. (F) Diedel knock-down (UAS-Die RNAi) in the fat body (PplGal4) leads to premature intestinal stem cell hyperproliferation (measured by quantification of pH3-positive cells [mitoses per gut] in young [10 d] and old [30 d] flies; bars represent mean ± SE, *n* = 20–25) compared to PplG4>+(w^1118^) controls. (G) Induction of Diedel (UAS-Diedel) or inhibition of Diedel (UAS-Die^RNAi^) specifically in the fat body (CGG4) or thoracic muscle (Act88FG4) has no effect on feeding behavior (measured by CAFE assay at 10 d of age, bars represents mean ± SE, *n* = 10). (H-I) Diedel overexpression (Act88FGal4>UAS-Diedel) inhibits some, but not all, IMD/NFkB pathway target genes (such as Dpt and Att but not Drs) and is correlated with a decrease in age-related commensal dysbiosis. (H) qRT-PCR of Drs, Dipt, and Att from young (10 d) and old (30 d) dissected midguts of flies with indicated genotypes; bars represent mean ± SE, *n* = 4; compared with Act88FG4>+(w^1118^) controls. (I) A 1/1,000 dilution of bacteria from dissected midguts from 30-d-old control flies (Act88G4>+[w^1118^]) or Act88FG4>UAS-Diedel flies plated on mannitol agar plates, 1 midgut per plate. Underlying data can be found in S1 Data. Att, Attacin A; Dpt, Diptericin; Drs, Drosomycin; IMD/NFkB, immune deficiency/nuclear factor kappa B; qRT-PCR, quantitative real-time PCR; RNAi, RNA interference.
(TIF)

S6 Fig Diedel protein conservation and function. **Related to** Figs 4 and 5. (A) Phylogeny of *Drosophila* Diedel (blue) identified in the genome of the indicated viruses (green). (B-C) Diedel is a secreted protein. Protein extracted from S2 cells transfected with empty plasmid (pAC5.1 V5-His) or a plasmid expressing tagged Diedel (Die^V5^), tagged ORF121 (ORF121^V5^), or tagged Recombinant ORF 121 (Rec-ORF121^V5^) was analyzed by western blot with anti-V5 antibody in either (B) cell lysate or (C) supernatant (conditioned media). (D-E) Protein extracted from S2 cells untransfected (Mock) or transfected with empty plasmid (pAC5.1), a plasmid expressing tagged Diedel (Die^V5^), tagged Upd1 (Upd1^V5^), tagged Upd2 (Upd2^V5^), or tagged Upd3 (Upd3^V5^) were analyzed by western blot with anti-V5 antibody in (D) cell lysate and (E) supernatant. (F-H) *Drosophila* S2 cells exposed to UV (100 mJ/cm^2^) and treated with mock or Diedel-V5 (+Die^V5^) conditioned media (F) Att, (G) Dipt, (H) Drs transcription (measured by qRT-PCR); bars represent mean ± SE, *n* = 4. (I-J) dsRNA targeted against RFP (control) or Diedel (“Die”) in S2 Cells exposed to UV (100 mJ/cm^2^) (I) Att and (J) Dipt measured by qRT-PCR; bars represent mean ± SE, *n* = 3. Underlying data can be found in S1 Data. Att, Attacin A; Dipt, Diptericin; Drs, Drosomycin; dsRNA, double-stranded RNA; qRT-PCR, quantitative real-time PCR; RFP, red fluorescent protein; Upd1, unpaired 1; Upd2, unpaired 2; Upd3, unpaired 3.
(TIF)

S7 Fig Fat body–derived Diedel governs age-related intestinal apoptosis. **Related to** Fig 6. (A) Cas-3 immunostaining occasionally revealed nuclear Cas-3 staining in intestinal enterocytes (Cas-3 [green], armadillo [red], and DAPI [blue]). Nuclear-positive cells were not considered in final analysis. (B-C) TUNEL-staining analysis in the midgut. (B) Extended examples of TUNEL staining specifically in the posterior midgut during aging (TUNEL [green] and DAPI [blue]). (C) Image showing unspecific TUNEL staining in the gastric region (and the area immediately posterior) of the midgut. This region was excluded from all analysis of TUNEL immunostaining. (D-E) CGGal4>UAS-Die^RNAi^ flies display elevated apoptosis at earlier time points during aging (20 d of age), monitored by Cas-3 immunostaining and TUNEL immunostaining in the posterior midgut compared with CGG4>+(w^1118^) and CGGal4>UAS-Diedel controls. (D) Representative images of Cas-3 immunostaining—Cas-3 (green), armadillo (red), and DAPI (blue)—(E) and TUNEL immunostaining (green), DAPI (blue). (F-H) PplGal4>UAS-Die^RNAi^ flies display elevated apoptosis at earlier time points during aging (30 d of age) monitored by Cas-3 immunostaining and TUNEL immunostaining in the posterior midgut compared to PplG4>+(w^1118^) and PplGal4>UAS-Diedel controls. (F) Quantification of TUNEL immunostaining in the posterior midgut of flies with the indicated genotype; represented as dot plot (average and SE, red line); *n* = 5–10. (G) Representative images of TUNEL immunostaining (green), DAPI (blue), and (H) Cas-3 immunostaining; Cas-3 (green), armadillo (Red), and DAPI (blue). Underlying data can be found in S1 Data. Cas-3, caspase 3.
(TIF)

S8 Fig Diedel attenuates JAK/STAT pathway activity by inhibiting Upd3 transcriptional induction. **Related to** Fig 6. (A-B) S2 cells exposed to UV (100 mJ/cm^2^) and Diedel-V5 conditioned media (+Die^V5^) display an inhibition in JAK/STAT pathway activation. (A) Inhibition of Upd3 transcription and (B) Socs36e transcription (measured by qRT-PCR, bars represent mean ± SE, *n* = 4) compared with cells exposed to mock-conditioned media (controls). (C) dsRNA targeting of Diedel (“Die”) in S2 cells leads to an increase in UV-induced (100 mJ/cm^2^) Upd3 transcription (measured by qRT-PCR, compared with control [dsRNA RFP]; bars represent mean ± SE, *n* = 3). (D) Schematic representation of Diedel action: Diedel inhibits apoptosis, which prevents Upd3 expression, leading to JAK/STAT pathway attenuation. (E) Induction of apoptosis in S2 cells leads to increases in Upd3 and Dronc transcription and JAK/STAT pathway activation (Soc36e) without JNK activation (Puc). dsRNA targeting RFP (Mock) or Diap1 in S2 cells; transcription of indicated genes measured by qRT-PCR, bars represent mean ± SE, *n* = 3. (F) Diedel does not attenuate JAK/STAT pathway activity directly (through influencing Upd/receptor binding). JAK/STAT pathway activation time course evaluated by Socs36e transcription measured by qRT-PCR in S2 cells exposed to indicated conditioned media; bars represent mean ± SE, *n* = 4. (G-J) Systemic Diedel inhibits JAK/STAT pathway activity in vivo, in the intestine, after *Ecc15* infection and during aging by inhibiting Upd3 expression. JAK/STAT activation was evaluated with 10xSTAT GFP flies; (G) Act88FG4>UAS-Diedel, 10xSTAT GFP flies display an attenuation of JAK/STAT pathway activation 18 h after *Ecc15* ingestion compared to controls (Act88G4>+[w^1118^], 10XSTAT-GFP). Representative images of posterior midgut; 10XSTAT-GFP (green), Armadillo (red), DAPI (blue). (H) Upd3 relative expression in dissected midguts of flies with indicated genotypes (measured by qRT-PCR, bars represent mean ± SE, *n* = 4). (G) Act88FG4>UAS-Diedel, 10xSTAT-GFP flies display an attenuation of JAK/STAT pathway during aging (30 d of age) compared to controls (Act88G4>+[w^1118^], 10XSTAT-GFP). Representative images of posterior midgut; 10XSTAT-GFP (green), Armadillo (red), DAPI (blue). (H) Upd3 relative expression in dissected midguts of flies with indicated genotypes at indicated ages (measured by qRT-PCR, bars represent mean ± SE, *n* = 4). (K) Inhibition of UV-induced proinflammatory cytokine by Diedel (conditioned media) in MEFs. IL-1β and TNFα transcription (measured by qRT-PCR at indicated time points after UV treatment, bars represent mean ± SE, *n* = 4), compared with controls exposed to mock-conditioned media. Underlying data can be found in S1 Data. Diap1, *Drosophila* inhibitor of apoptosis 1; Dronc, death regulator Nedd2-like caspase; dsRNA, double-stranded RNA; *Ecc15*, *Erwinia carotovora carotovora 15*; IL-1β, interleukin beta; JAK/STAT, Janus kinase/signal transducer and activator of transcription proteins; JNK, Jun-N-terminal kinase; MEF, mouse embryonic fibroblast; Puc, Puckered; qRT-PCR, quantitative real-time PCR; RFP, red fluorescent protein; Socs36e, suppressor of cytokine signaling 36E; TNFα, tumor necrosis factor alpha; Upd3, unpaired 3.
(TIF)

S1 Table Table summarizing parameters and statistics for Gal4 lifespan analysis (related to Figs 1–3). (TIF)

S2 Table Table summarizing parameters and statistics for RU486-dependent lifespan analysis (related to Figs 1–3). RU486, mifepristone.
(TIF)

S3 Table Table summarizing parameters and statistics for TubGS dose-dependent RU486 lifespan analysis (related to Fig 3). RU486, mifepristone.
(TIF)

S4 Table Primer sequences (related to Figs 2–6). (TIF)

S1 Data Raw data. (XLSX)

S2 Data Complete lifespan data summarizing parameters and statistics for all individual experiments. (XLSX)

## Abbreviations

Cas-3: caspase 3
Cas-9: caspase 9
Dcp-1: death caspase 1
Dronc: death regulator Nedd2-like caspase
dsRNA: double-stranded RNA
*Ecc15*: *Erwinia carotovora carotovora 15*
ER stress: endoplasmic reticulum stress response
FRT: flippase recombination target
GO: Gene Ontology
Hid: head involution defect
IAP: inhibitor of apoptosis
IGF: insulin-like growth factor
IL-1β: interleukin 1 beta
ISC: intestinal stem cells
Jak/Stat: Janus kinase/signal transducer and activator of transcription proteins
JNK: Jun-N-terminal kinase
MEF: mouse embryonic fibroblast
MOI: multiplicity of infection
mtDNA: mitochondrial DNA
NFkB: nuclear factor kappa B
PBST: PBS containing 0.1% Triton X-100
Puc: Puckered
qRT-PCR: quantitative real-time PCR
RFP: red fluorescent protein
RNAi: RNA interference
RPKM: reads per kbp per million reads
Rpr: Reaper
RU486: mifepristone
SfAV-1a: *Spodoptera frugiperda* Ascovirus 1a
TNFα: tumor necrosis factor alpha
TOR: target of rapamycin
UAS: upstream activating sequence
Upd3: unpaired 3
UPR: unfolded protein response
UVC: ultraviolet C light
VSV: vesicular stomatitis virus
