# Supplementary figures and images for: A virus-acquired host cytokine controls systemic aging by antagonizing apoptosis
Source: PLoS Biol. 2018 Jul 23;16(7):e2005796. doi: 10.1371/journal.pbio.2005796 (PMC6072105; doi:10.1371/journal.pbio.2005796)

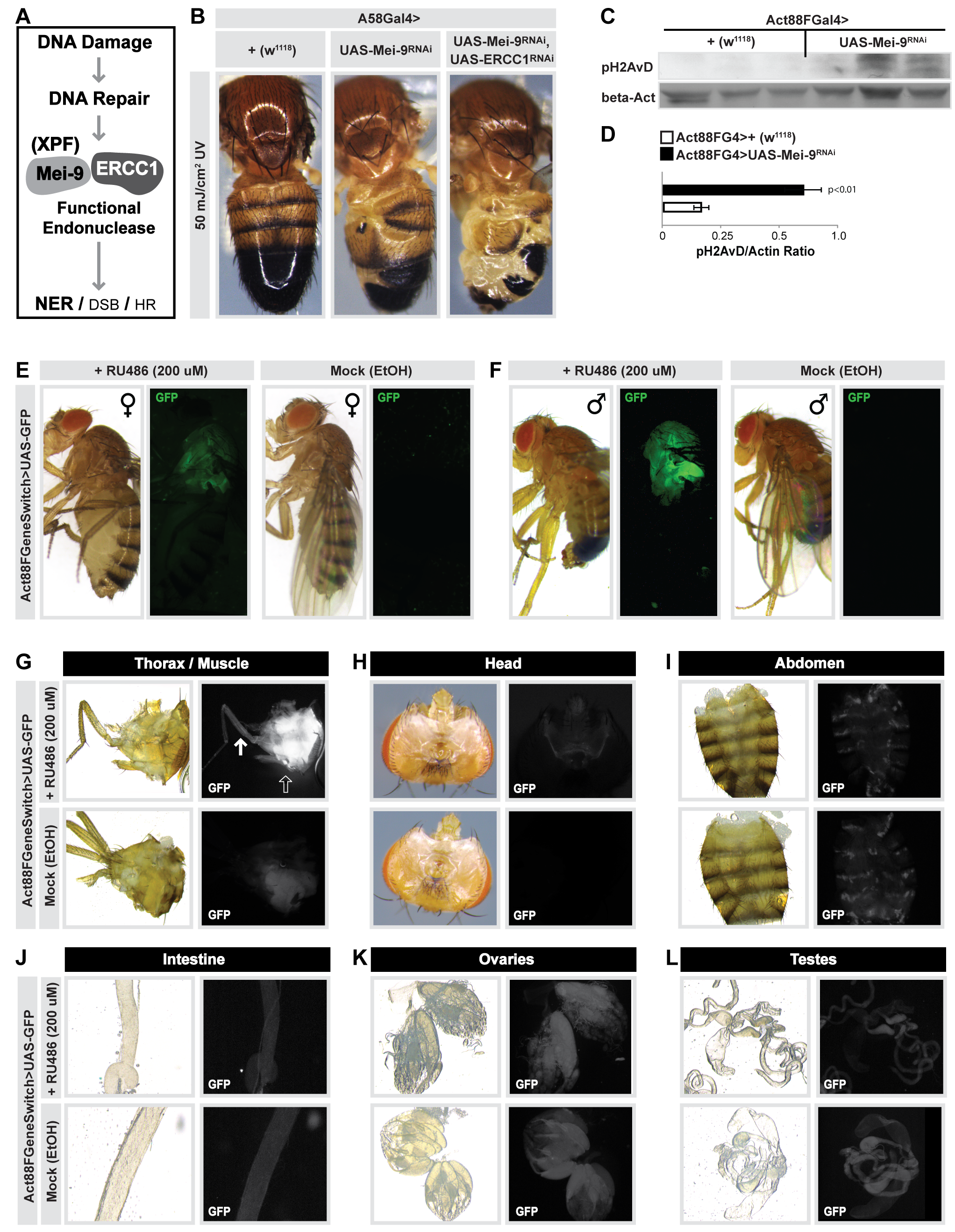

Supplement: S1 Fig — Related to Fig 1. (A-D) Mei-9 and ERCC1 heterodimers constitute a conserved DNA-repair endonuclease involved in NER pathway. (B) Silencing Mei-9 (UAS-Mei-9 RNAi) or Mei-9 and ERRC1 concurrently (UAS-Mei-9 RNAi, UAS-ERCC1 RNAi) in the larva epidermis (A58Gal4) inhibits DNA repair after UV irradiation. Images of adult abdominal cuticle after larval UV treatment. (C) Immunoblot of protein extract from dissected thorax of Act88FG4>+(w1118) controls (Day 10) and Act88FG4>UAS-Mei-9 RNAi (Day 10) flies analyzed by western blotting with pH2AvD (normalized to beta-Actin [“beta-Act”]). (D) Western blot quantification; bars represent mean ± SE, n = 3. (E-L) Characterization of Act88FGeneSwitch driver specificity (utilizing UAS-nlsGFP). Act88FGS>UAS-GFP flies were fed for 5 d with mock-treated food (80% ethanol control) or RU486-treated food (200 uM) to induce GFP expression. Act88FGS>UAS-GFP flies display RU486-dependent GFP expression specifically in the thoracic skeletal muscle—including the longitudinal IFMs, dorsal lateral muscles, dorsal ventral muscles—and weaker expression in leg muscles. No GFP expression is observed in any tissue in the carcass or head. Representative images of (E) whole female (F) or male flies’ (G) dissected thorax; (H) head; (I) abdomen; (J) intestine; (K) ovaries; and (L) testes. Underlying data can be found in S1 Data. GFP, green fluorescent protein; IFM, indirect flight muscle; NER, nucleotide excision repair; pH2AvD, phospo-Histone 2A gamma; RNAi, RNA interference; RU486, mifepristone. (TIF) [file pbio.2005796.s001.tif]

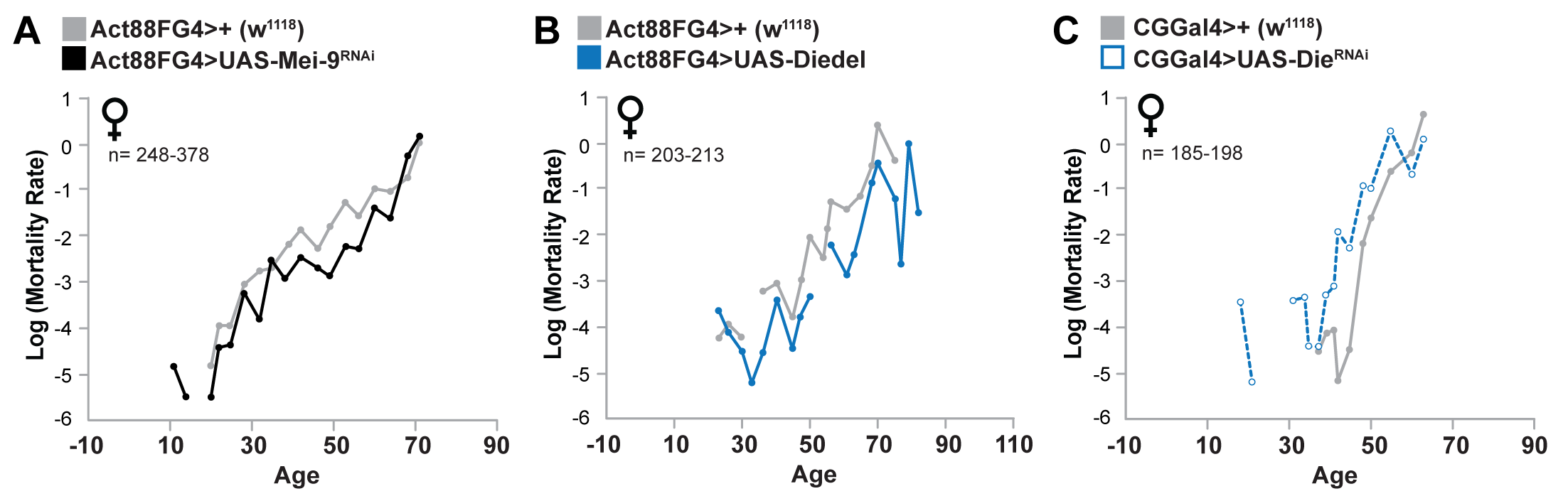

Supplement: S2 Fig — Related to Figs 1, 2 and 3. (A-B) Mortality plots (female flies) associated with mu-specific (A) inhibition of Mei-9 (UAS-Mei-9 RNAi) using the Act88FGal4 driver (compared to Act88FG4>+[w1118] controls) and (B) overexpression of Diedel using the Act88FGal4 driver (compare to Act88FG4>+[w1118] controls). (C) Mortality plots (female flies) associated with fat body specific inhibition of Diedel (UAS-Die RNAi using the CGGal4 driver, compared with CGG4>+[w1118] controls). These plots correspond to survival analysis found in Figs 1C, 2G and 3G respectively. Underlying data can be found in S1 Data. mu-specific, muscle-specific; RNAi, RNA interference. (TIF) [file pbio.2005796.s002.tif]

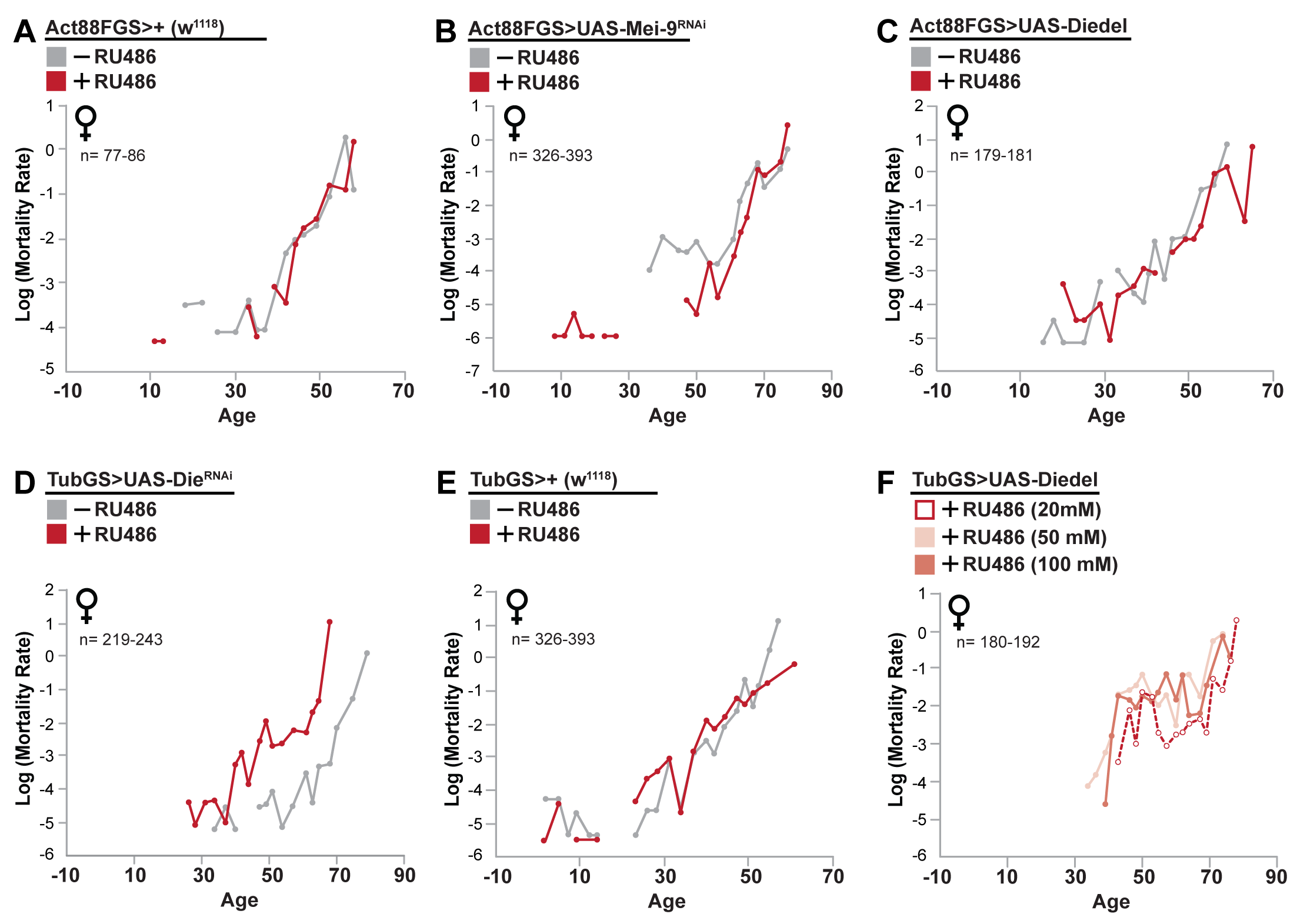

Supplement: S3 Fig — Related to Figs 1–3. (A-C) Mortality plots (female flies) associated with the mu-specific GeneSwitch inducible driver (Act88FGS) (A) Act88GS>+(w1118) +RU486 compared with −RU486 (vehicle alone) sibling controls; (B) Act88FGS>UAS-Mei-9RNAi +RU486 compared with −RU486 (vehicle alone) sibling controls; (C) Act88FGS>UAS-Diedel +RU486 compared with −RU486 (vehicle alone) sibling controls. These plots correspond to survival analyses found in S2 Table, Figs 1D and 2H, respectively. (D-F) Mortality plots (female flies) associated with ubiquitous GeneSwitch inducible driver (Tubulin(“Tub”)GS, +RU486 compared with −RU486 [vehicle alone] sibling controls). (D) TubGS>UAS-DieRNAi. (E) TubGS>+(w1118). (F) RU486 dose dependency of mortality in TubGS>Diedel female flies, utilizing 20 mM, 50 mM, and 100 mM doses of RU486. These plots correspond to survival analyses found in Fig 3H, S2 Table, and Fig 3J/S2 Table, respectively. Underlying data can be found in S1 Data. mu-specific, muscle-specific; RU486, mifepristone. (TIF) [file pbio.2005796.s003.tif]

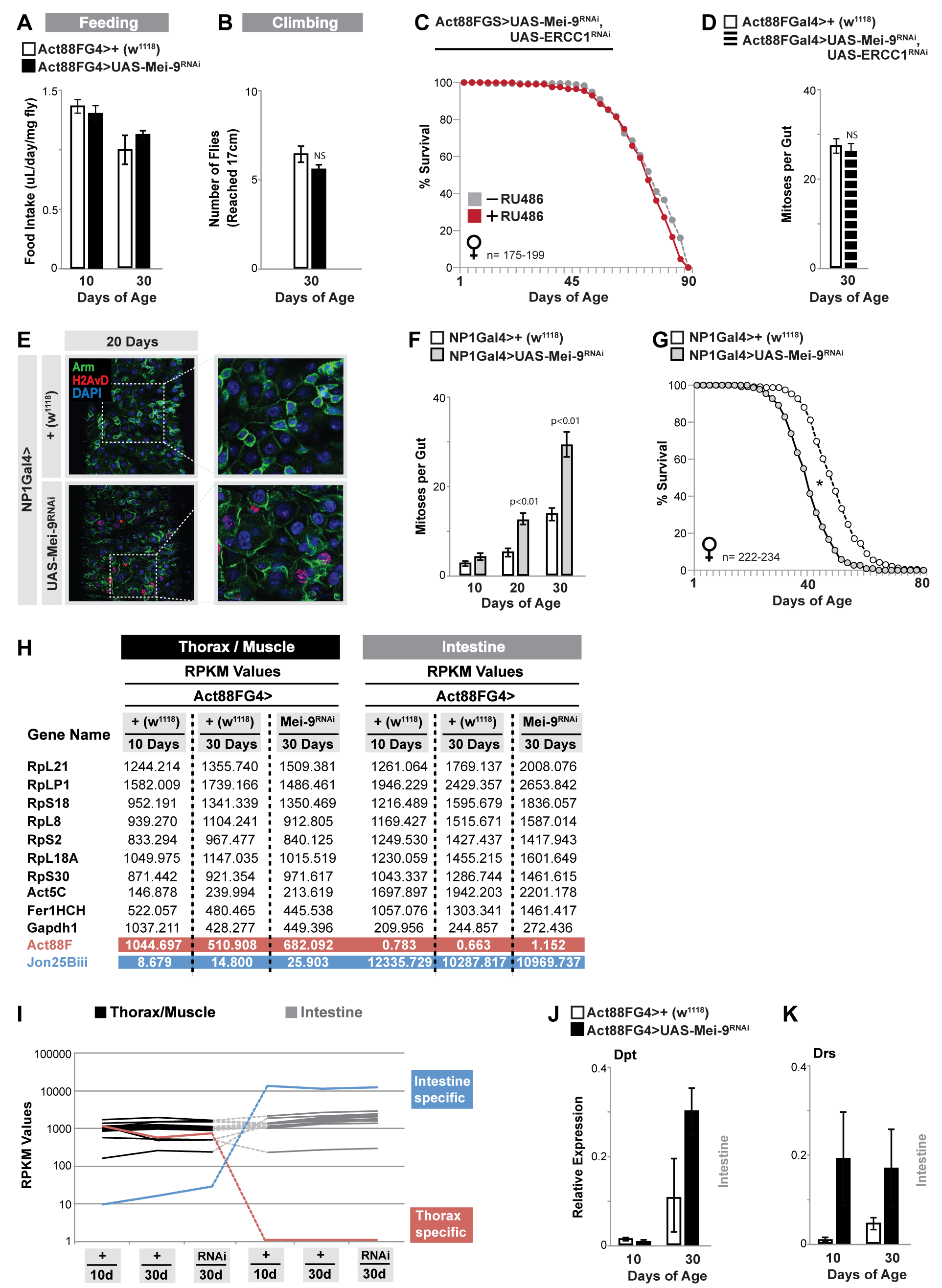

Supplement: S4 Fig — Related to Figs 1 and 2. (A-B) Knock-down of Mei-9 (UAS-Mei-9 RNAi) specifically in the adult thoracic muscle (Act88FGal4) has no effect on (A) feeding behavior (measured by CAFE assay, bars represent mean ± SE, n = 4 independent samples) or (B) climbing (bars represent mean ± SE, n = 5 cohorts of 20 flies) compared with Act88FG4>+(w1118) controls. (C-D) Knock-down of Mei-9 and ERRC1 concurrently (UAS-Mei-9 RNAi, UAS-ERCC1 RNAi) specifically in thoracic muscle has no effect on (C) lifespan (survival curves, S2 Table) or (D) age-related intestinal stem cell hyperproliferation (quantified by pH3-positive cells [mitoses per gut] at 30 d, bar represents mean ± SE, n = 25–30) compared to Act88FG4>+(w1118) controls. (E-F) Knock-down of Mei-9 (UAS-Mei-9 RNAi) specifically in intestinal enterocytes (NP1Gal4) leads to tissue-autonomous (E) accumulation of DNA damage (measured by immunostaining with pH2AvD antibody in dissected midguts), (F) increases in intestinal stem cell mitoses (quantified by pH3-positive cells at day 10, 20, and 30; bars represent mean ± SE, n = 25–30), and (G) decreases in lifespan (survival curves, S1 Table) compared with NP1G4>+(w1118) controls. (H-I) (H) RPKM values for select, basally high genes that show no change upon muscle-specific depletion of MEI-9 in all of the unique tissue transcriptomes (thorax/muscle and intestine). Plotted on graph (I); log scale; each line represents a unique gene. (J-K) Intestinal immune gene induction during aging in Act88FG4>UAS-Mei-9RNAi flies, (J) Diptericin (“Dpt”) and (K) Drosomycin (“Drs”) measured in dissected midguts from young (10 d) and old (30 d) flies by qRT-PCR; bars represent mean ± SE, n = 3; compared with Act88FG4>+(w1118) controls. Underlying data can be found in S1 Data. pH2AvD, phospho-Histone 2A gamma; pH3, phospho-Histone H3; qRT-PCR, quantitative real-time PCR; RNAi, RNA interference; RPKM, reads per kbp per million reads. (TIF) [file pbio.2005796.s004.tif]

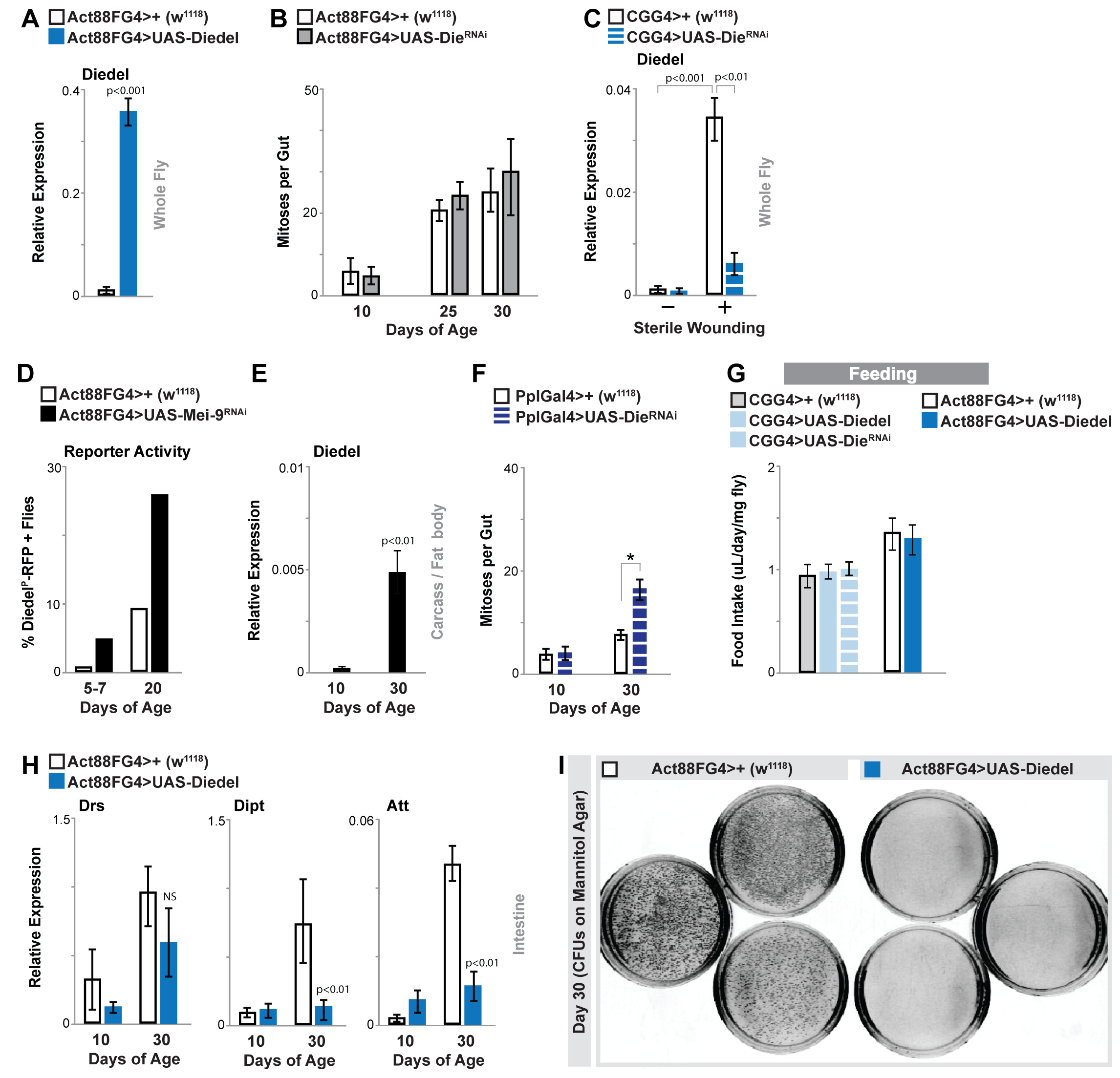

Supplement: S5 Fig — Related to Figs 2 and 3. (A) Diedel transcription (measured by qRT-PCR) in Act88fG4>UAS-Diedel whole flies; bars represent mean ± SE, n = 3; compared with Act88FG4>+(w1118) controls. (B) Diedel knock-down (UAS-Die RNAi) specifically in the thoracic muscle (Act88FGal4) has no effect on intestinal stem cell proliferation during aging (measured by number of pH3-positive cells at day 10, 25, and 30; bars represent mean ± SE; n = 25–30) compared to Act88FG4>+(w1118) controls. (C) UAS-Diedel RNAi efficiency was evaluated in the fat body after sterile wounding. Diedel is induced after sterile wounding in the control flies (CGG4>+), and this induction is inhibited in CGG4>UAS-DieRNAi animals; bars represent mean ± SE; n = 3; whole flies were used in this analysis. (D) Knock-down of Mei-9 (UAS-Mei-9 RNAi) specifically in thoracic muscle (Act88FGal4) induces accelerated and age-related activation of a Diedel reporter; displayed by an increase in the proportion of Diedel-RFP-positive flies (DiedelP-RFP +) in Act88FG4>UAS-MEI9RNAi animals compared with Act88FG4>+(w1118) controls. (E) Knock-down of Mei-9 (UAS-Mei-9 RNAi) specifically in thoracic muscle (Act88FGal4) promotes elevated Diedel transcription in the carcass/fat body during aging (measured by qRT-PCR of dissected carcass in young [10 d] and old [30 d] flies; bars represent mean ± SE, n = 3) compared with Act88FG4>+(w1118) controls. (F) Diedel knock-down (UAS-Die RNAi) in the fat body (PplGal4) leads to premature intestinal stem cell hyperproliferation (measured by quantification of pH3-positive cells [mitoses per gut] in young [10 d] and old [30 d] flies; bars represent mean ± SE, n = 20–25) compared to PplG4>+(w1118) controls. (G) Induction of Diedel (UAS-Diedel) or inhibition of Diedel (UAS-DieRNAi) specifically in the fat body (CGG4) or thoracic muscle (Act88FG4) has no effect on feeding behavior (measured by CAFE assay at 10 d of age, bars represents mean ± SE, n = 10). (H-I) Diedel overexpression (Act88FGal4>UAS-D [file pbio.2005796.s005.tif]

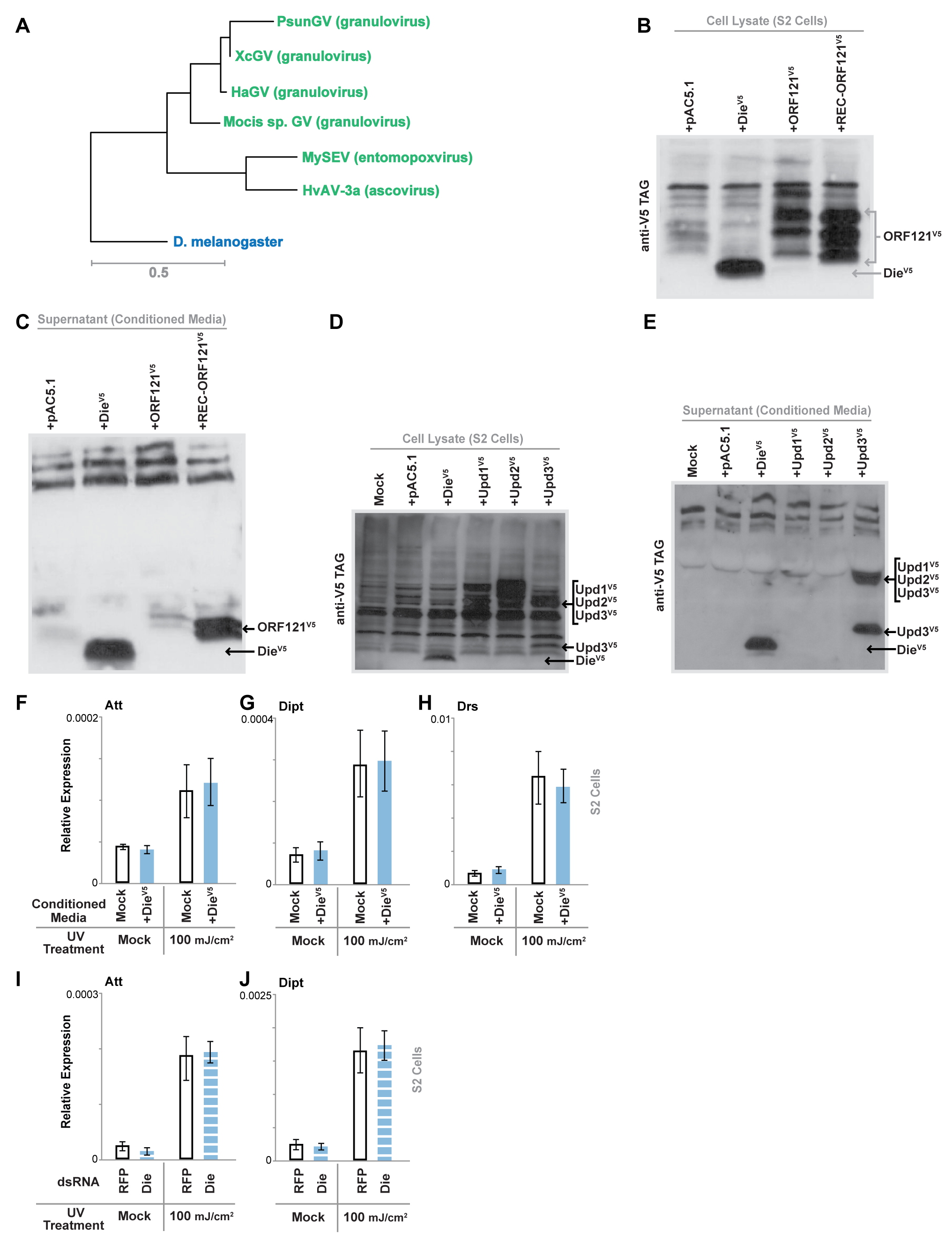

Supplement: S6 Fig — Related to Figs 4 and 5. (A) Phylogeny of Drosophila Diedel (blue) identified in the genome of the indicated viruses (green). (B-C) Diedel is a secreted protein. Protein extracted from S2 cells transfected with empty plasmid (pAC5.1 V5-His) or a plasmid expressing tagged Diedel (DieV5), tagged ORF121 (ORF121V5), or tagged Recombinant ORF 121 (Rec-ORF121V5) was analyzed by western blot with anti-V5 antibody in either (B) cell lysate or (C) supernatant (conditioned media). (D-E) Protein extracted from S2 cells untransfected (Mock) or transfected with empty plasmid (pAC5.1), a plasmid expressing tagged Diedel (DieV5), tagged Upd1 (Upd1V5), tagged Upd2 (Upd2V5), or tagged Upd3 (Upd3V5) were analyzed by western blot with anti-V5 antibody in (D) cell lysate and (E) supernatant. (F-H) Drosophila S2 cells exposed to UV (100 mJ/cm2) and treated with mock or Diedel-V5 (+DieV5) conditioned media (F) Att, (G) Dipt, (H) Drs transcription (measured by qRT-PCR); bars represent mean ± SE, n = 4. (I-J) dsRNA targeted against RFP (control) or Diedel (“Die”) in S2 Cells exposed to UV (100 mJ/cm2) (I) Att and (J) Dipt measured by qRT-PCR; bars represent mean ± SE, n = 3. Underlying data can be found in S1 Data. Att, Attacin A; Dipt, Diptericin; Drs, Drosomycin; dsRNA, double-stranded RNA; qRT-PCR, quantitative real-time PCR; RFP, red fluorescent protein; Upd1, unpaired 1; Upd2, unpaired 2; Upd3, unpaired 3. (TIF) [file pbio.2005796.s006.tif]

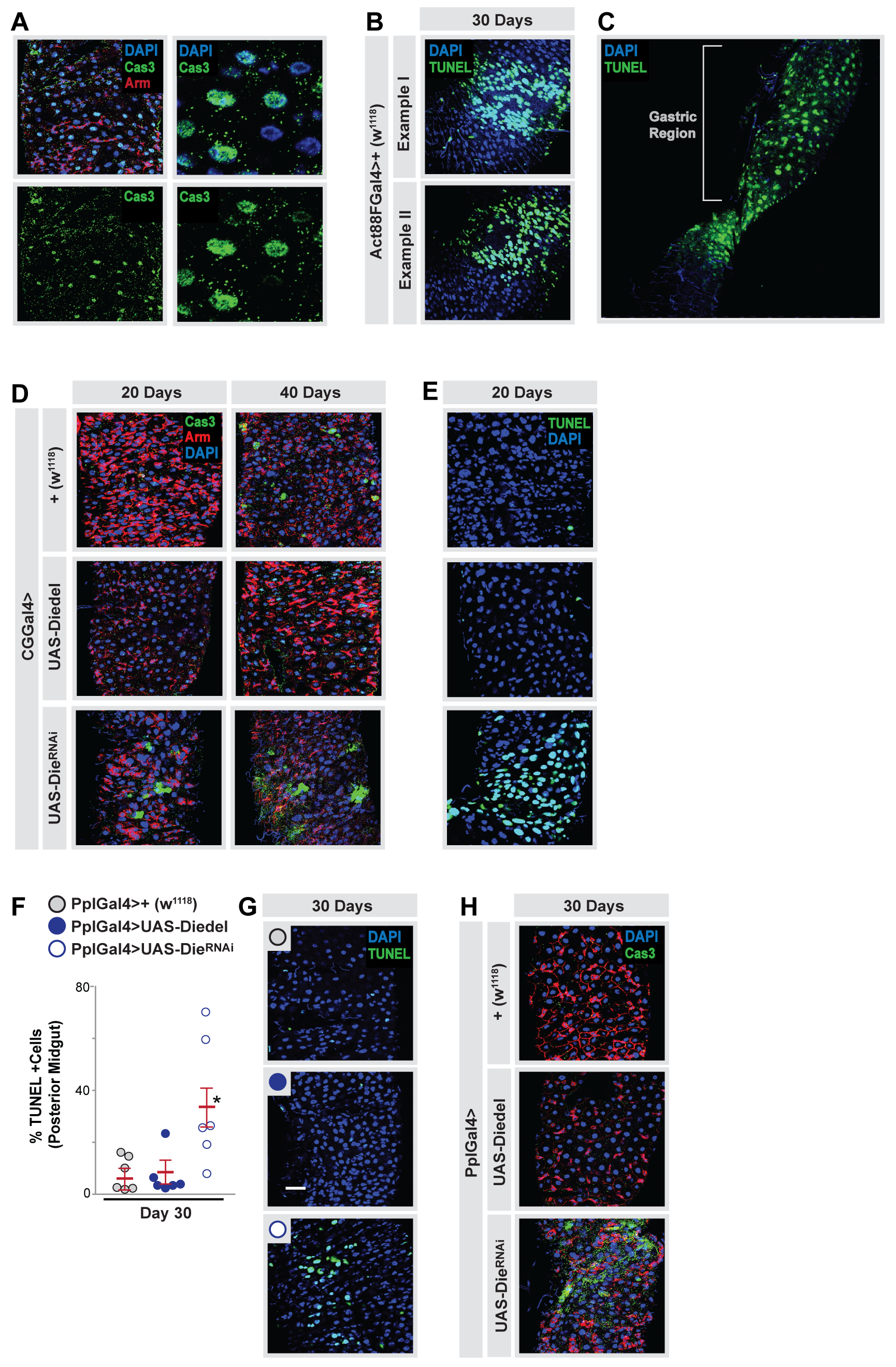

Supplement: S7 Fig — Related to Fig 6. (A) Cas-3 immunostaining occasionally revealed nuclear Cas-3 staining in intestinal enterocytes (Cas-3 [green], armadillo [red], and DAPI [blue]). Nuclear-positive cells were not considered in final analysis. (B-C) TUNEL-staining analysis in the midgut. (B) Extended examples of TUNEL staining specifically in the posterior midgut during aging (TUNEL [green] and DAPI [blue]). (C) Image showing unspecific TUNEL staining in the gastric region (and the area immediately posterior) of the midgut. This region was excluded from all analysis of TUNEL immunostaining. (D-E) CGGal4>UAS-DieRNAi flies display elevated apoptosis at earlier time points during aging (20 d of age), monitored by Cas-3 immunostaining and TUNEL immunostaining in the posterior midgut compared with CGG4>+(w1118) and CGGal4>UAS-Diedel controls. (D) Representative images of Cas-3 immunostaining—Cas-3 (green), armadillo (red), and DAPI (blue)—(E) and TUNEL immunostaining (green), DAPI (blue). (F-H) PplGal4>UAS-DieRNAi flies display elevated apoptosis at earlier time points during aging (30 d of age) monitored by Cas-3 immunostaining and TUNEL immunostaining in the posterior midgut compared to PplG4>+(w1118) and PplGal4>UAS-Diedel controls. (F) Quantification of TUNEL immunostaining in the posterior midgut of flies with the indicated genotype; represented as dot plot (average and SE, red line); n = 5–10. (G) Representative images of TUNEL immunostaining (green), DAPI (blue), and (H) Cas-3 immunostaining; Cas-3 (green), armadillo (Red), and DAPI (blue). Underlying data can be found in S1 Data. Cas-3, caspase 3. (TIF) [file pbio.2005796.s007.tif]

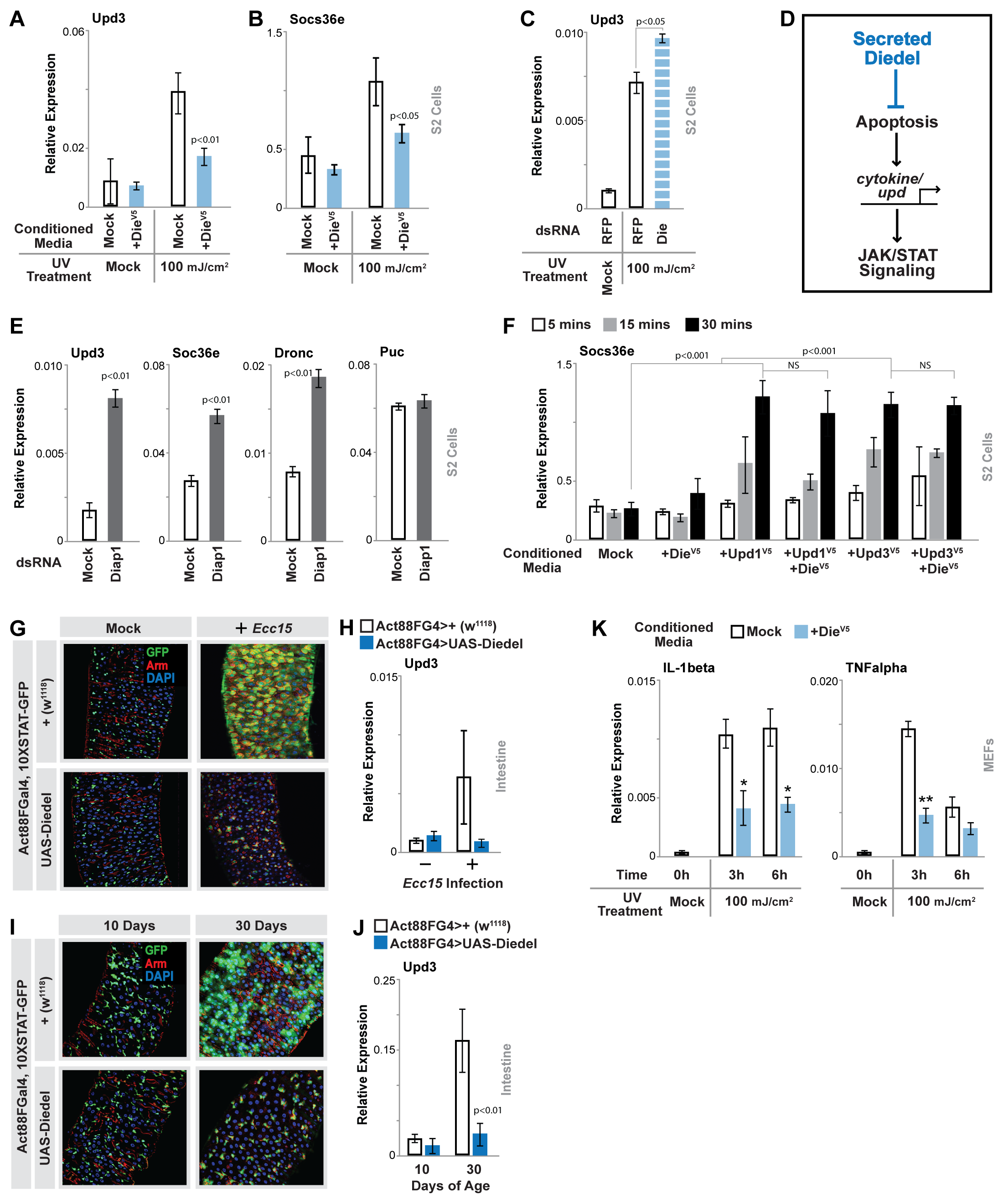

Supplement: S8 Fig — Related to Fig 6. (A-B) S2 cells exposed to UV (100 mJ/cm2) and Diedel-V5 conditioned media (+DieV5) display an inhibition in JAK/STAT pathway activation. (A) Inhibition of Upd3 transcription and (B) Socs36e transcription (measured by qRT-PCR, bars represent mean ± SE, n = 4) compared with cells exposed to mock-conditioned media (controls). (C) dsRNA targeting of Diedel (“Die”) in S2 cells leads to an increase in UV-induced (100 mJ/cm2) Upd3 transcription (measured by qRT-PCR, compared with control [dsRNA RFP]; bars represent mean ± SE, n = 3). (D) Schematic representation of Diedel action: Diedel inhibits apoptosis, which prevents Upd3 expression, leading to JAK/STAT pathway attenuation. (E) Induction of apoptosis in S2 cells leads to increases in Upd3 and Dronc transcription and JAK/STAT pathway activation (Soc36e) without JNK activation (Puc). dsRNA targeting RFP (Mock) or Diap1 in S2 cells; transcription of indicated genes measured by qRT-PCR, bars represent mean ± SE, n = 3. (F) Diedel does not attenuate JAK/STAT pathway activity directly (through influencing Upd/receptor binding). JAK/STAT pathway activation time course evaluated by Socs36e transcription measured by qRT-PCR in S2 cells exposed to indicated conditioned media; bars represent mean ± SE, n = 4. (G-J) Systemic Diedel inhibits JAK/STAT pathway activity in vivo, in the intestine, after Ecc15 infection and during aging by inhibiting Upd3 expression. JAK/STAT activation was evaluated with 10xSTAT GFP flies; (G) Act88FG4>UAS-Diedel, 10xSTAT GFP flies display an attenuation of JAK/STAT pathway activation 18 h after Ecc15 ingestion compared to controls (Act88G4>+[w1118], 10XSTAT-GFP). Representative images of posterior midgut; 10XSTAT-GFP (green), Armadillo (red), DAPI (blue). (H) Upd3 relative expression in dissected midguts of flies with indicated genotypes (measured by qRT-PCR, bars represent mean ± SE, n = 4). (G) Act88FG4>UAS-Diedel, 10xSTAT-GFP flies display an attenuation of JAK/STAT pathway during [file pbio.2005796.s008.tif]

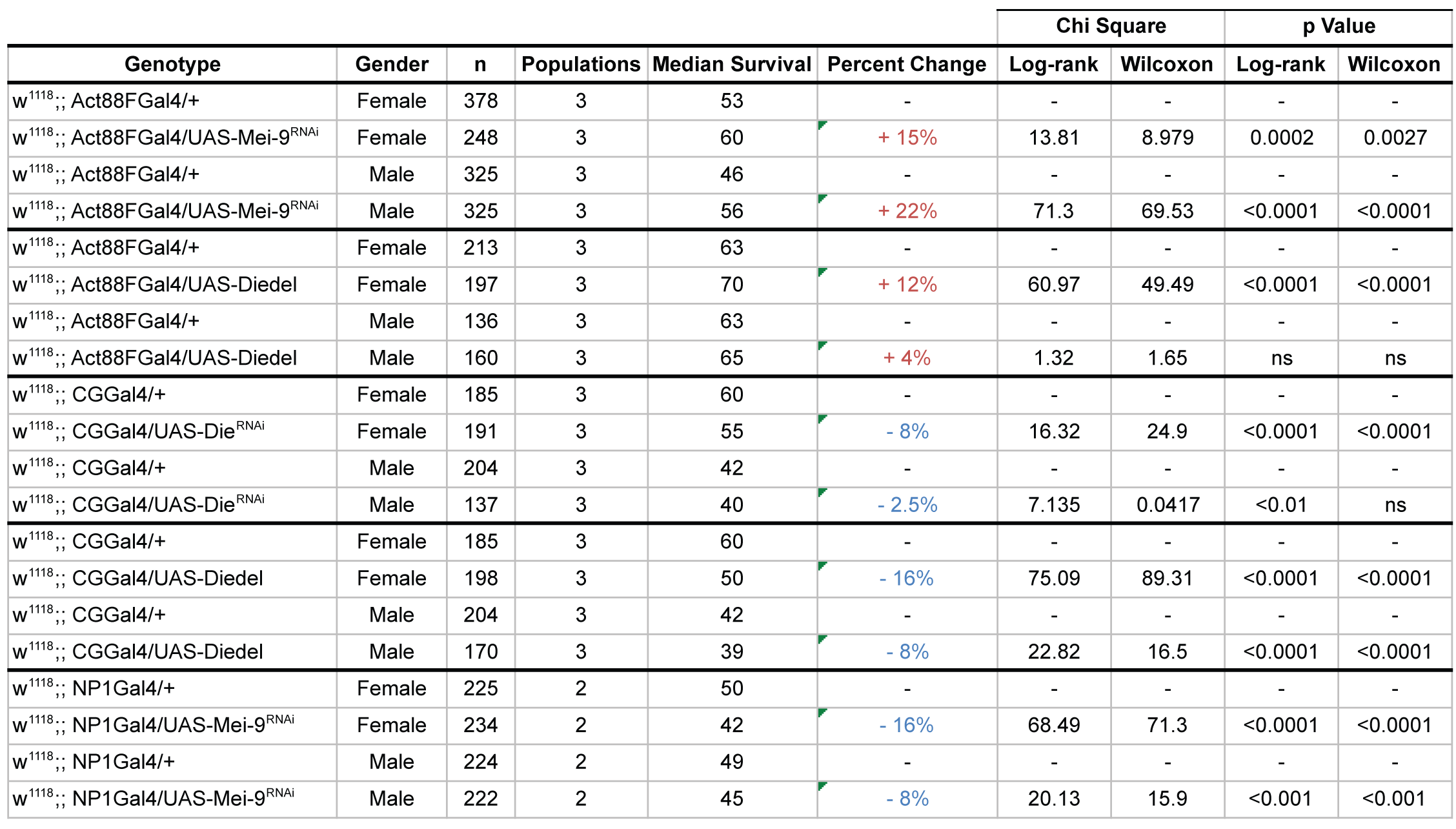

Supplement: S1 Table — (TIF) [file pbio.2005796.s009.tif]

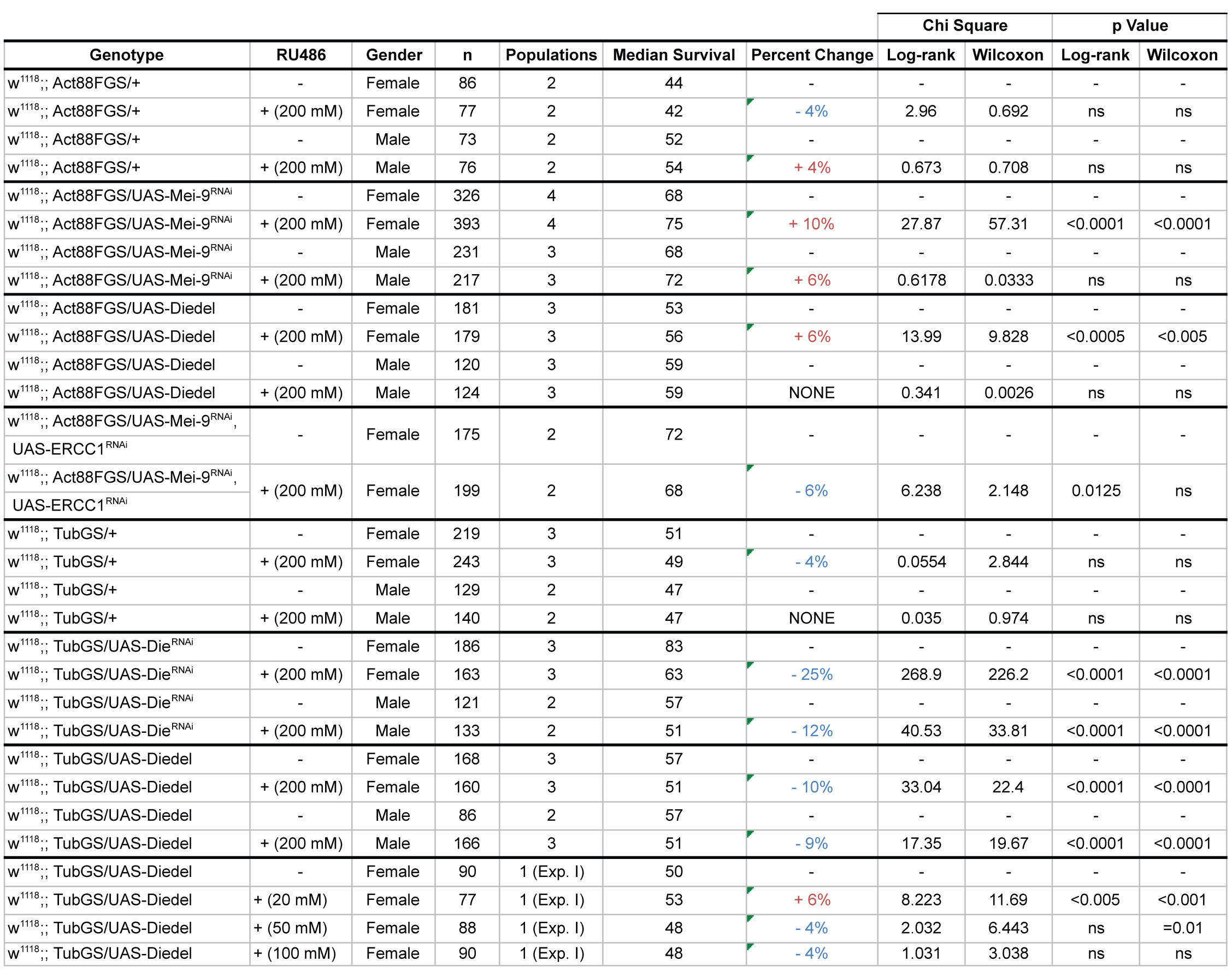

Supplement: S2 Table — RU486, mifepristone. (TIF) [file pbio.2005796.s010.tif]

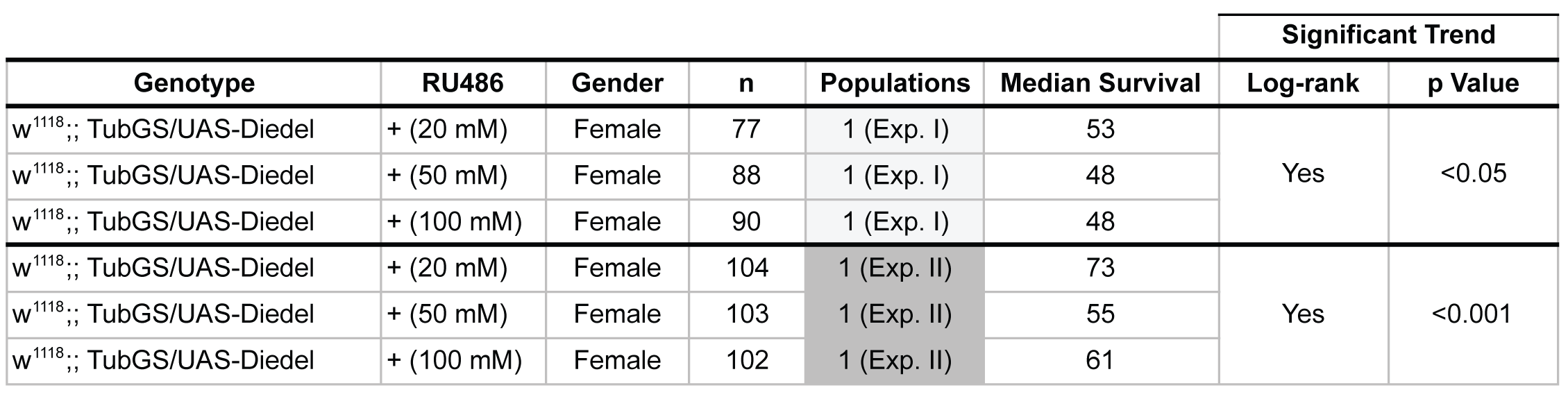

Supplement: S3 Table — RU486, mifepristone. (TIF) [file pbio.2005796.s011.tif]

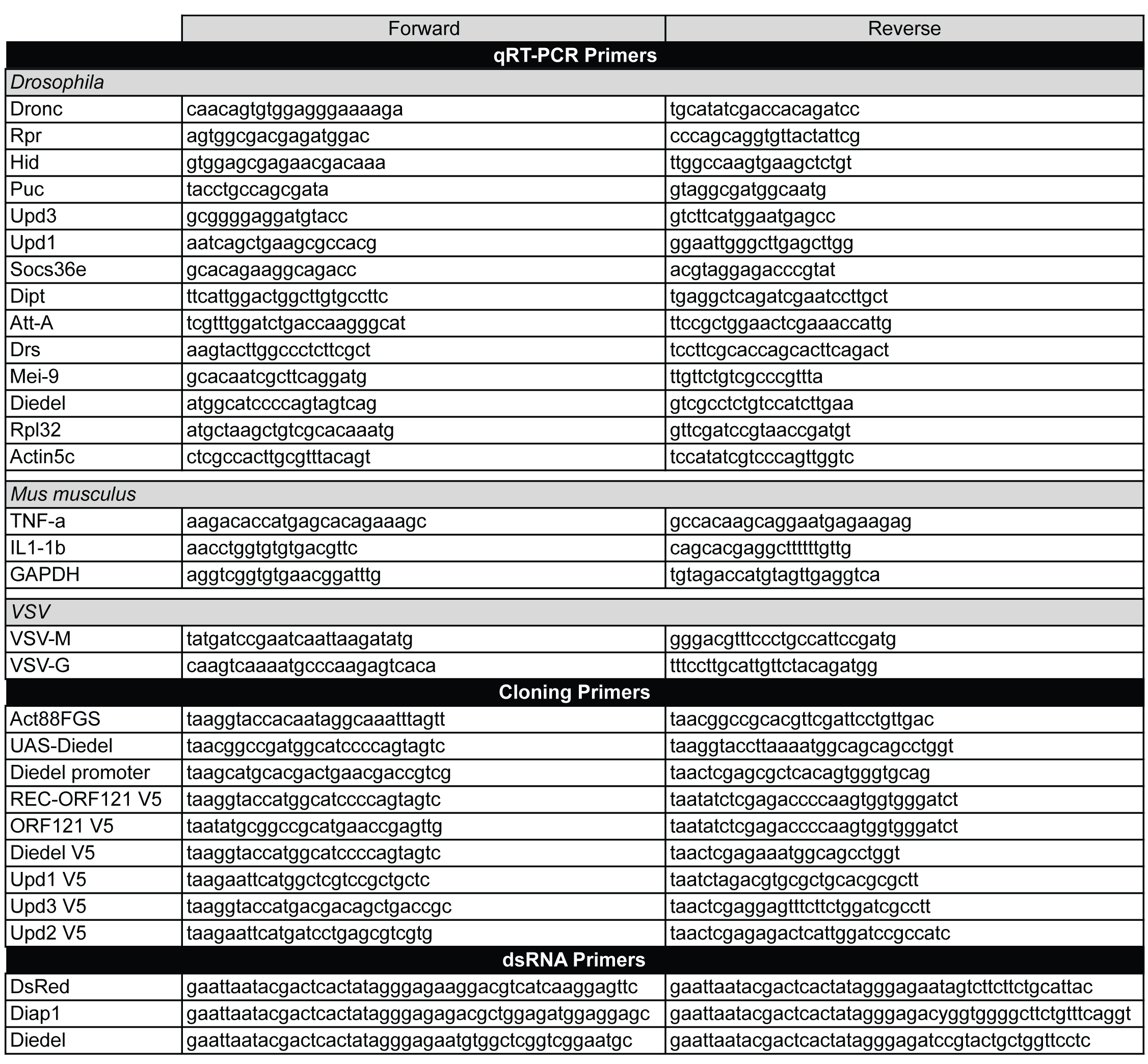

Supplement: S4 Table — (TIF) [file pbio.2005796.s012.tif]
